# Identification of multipotent drugs for COVID-19 therapeutics with the evaluation of their SARS-CoV2 inhibitory activity

**DOI:** 10.1016/j.csbj.2021.04.014

**Published:** 2021-04-07

**Authors:** Sugandh Kumar, Bharati Singh, Pratima Kumari, Preethy V. Kumar, Geetanjali Agnihotri, Shaheerah Khan, Tushar Kant Beuria, Gulam Hussain Syed, Anshuman Dixit

**Affiliations:** a Institute of Life Science, Nalco Square, Bhubaneswar, Odisha 751023, India; b School of Biotechnology, Kalinga Institute of Industrial Technology (KIIT) University, Bhubaneswar, Odisha 751024, India; c School of Chemical Technology, Kalinga Institute of Industrial Technology (KIIT) University, Bhubaneswar, Odisha 751024, India; d Regional Centre for Biotechnology (RCB), 3rd Milestone, Faridabad-Gurgaon, Haryana 121001, India

**Keywords:** Coronavirus, COVID-19, Drug repurposing, Network analysis, Docking, Polypharmacology, Molecular dynamics

## Abstract

The SARS-CoV2 is a highly contagious pathogen that causes COVID-19 disease. It has affected millions of people globally with an average lethality of ~3%. There is an urgent need of drugs for the treatment of COVID-19.

In the current studies, we have used bioinformatics techniques to screen the FDA approved drugs against nine SARS-CoV2 proteins to identify drugs for repurposing. Additionally, we analyzed if the identified molecules can also affect the human proteins whose expression in lung changed during SARS-CoV2 infection. Targeting such genes may also be a beneficial strategy to curb disease manifestation. We have identified 74 molecules that can bind to various SARS-CoV2 and human host proteins.

We experimentally validated our in-silico predictions using vero E6 cells infected with SARS-CoV2 virus. Interestingly, many of our predicted molecules viz. capreomycin, celecoxib, mefloquine, montelukast, and nebivolol showed good activity (IC50) against SARS-CoV2. We hope that these studies may help in the development of new therapeutic options for the treatment of COVID-19.

## Graphical abstract

**Figure:**
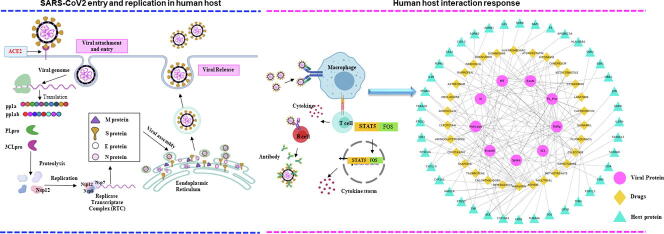

## 1 Introduction

Novel zoonotic viruses with the potential for rapid spread and significant pathology pose a grave threat to humans. During the last few decades many epidemics of viral diseases have occurred such as Ebola, Zika, Nipah, Avian influenza (H7N9), HIN1, Severe Acute Respiratory Syndrome Coronavirus 1 (SARS-CoV1), and Middle East Respiratory Syndrome Coronavirus (MERS-CoV) [1].

In the end of 2019 a highly infectious novel coronavirus, which was later renamed as severe acute respiratory syndrome coronavirus 2 (SARS-CoV2), emerged, which causes the disease termed “coronavirus disease-19” (COVID-19) [2]. COVID-19 has affected millions of people globally and the number is increasing alarmingly with the continuous surge in the number of cases in many countries world wide. COVID-19 is associated with an average lethality of ~3%. The elderly and those with comorbidities are at high risk of developing severe clinical manifestations. Infection with SARS-CoV2 results in acute respiratory distress syndrome (ARDS) leading to lung injury, respiratory distress, and lethality.. The extremely infectious nature of the disease, emergence of new hyperinfective strains, limited supply of vaccines and unavailability of effective and specific drugs is a serious cause of concern.

The SARS-CoV2, SARS-CoV1 and MERS-CoV belongs to the family of Coronaviridae and β-coronavirus genus [3]. While bats are considered to be the origin of SARS-CoV1 and SARS-CoV2, the intermediate host that led to human transmission of SARS-CoV2 is still unknown. Sequence analysis reveals that SARS-CoV2 is similar to coronavirus identified in Malayan pangolins (*Manis javanica*) [4]. The SARS-CoV2 genome is 29.8–29.9 kb positive‐sense single stranded RNA with 5′‐cap and 3′‐poly‐A tail. Its genome is organised into two segments that encode non-structural (Nsp) and structural proteins. The first segment is directly translated by ribosomal frameshifting into polyprotein 1a (486 kDa) or 1ab (790 kDa) (ORF1a, ORF1ab), which results in synthesis of non-structural proteins and formation of replication‐transcription complex (RTC) [5], [6]. The ORF1a/1ab covers two‐thirds of the whole genomic length and encodes for the 16 non-structural proteins (Nsp1‐16), which play critical roles in various viral processes. The discontinuous transcription of the second segment of viral genome results in formation of subgenomic RNAs (sgRNAs) containing common 5′‐ and 3′- leader and terminal sequences which serve as the template for subgenomic mRNA production [6]. The subgenomic mRNA’s encode for the 4 structural proteins (spike (S), membrane (M), envelope (E), and nucleocapsid (N) proteins) and 6 accessory proteins. [6]. The life cycle of SARS-CoV2 starts with its entry into the host cell through endocytosis initiated by its spike protein binding to the ACE2 receptor [7]. Subsequently, uncoating of the virus particle releases the genome, which is translated to generate replication‐transcription complex proteins. The viral RTC complex then generates full-length negative sense RNA that is subsequently transcribed into full-length genome. The viral genome and structural proteins are assembled into virions near the ER and Golgi interface and are transported out of the cell through vesicles by exocytosis [8].

The detailed understanding of the clinical manifestations and the underlying molecular mechanisms that drive disease pathogenesis are still unclear. There is no standard cure for the disease and the current COVID-19 therapeutic guidelines approved by FDA recommends the use of remdesivir and symptomatic treatment in hospitalized patients (www.covid19treatmentguidelines.nih.gov). Worldwide efforts to develop vaccines and drug against SARS-CoV2 are ongoing. While some of the vaccine candidates are being approved for use, however it will will take many months to validate their efficacy and safety in a large population. The ever evolving mutant strains also pose a risk of making the vaccine ineffective. Moreover, it has been estimated to take 2–3 years to vaccinate majority of the people on the globe. The ever-growing COVID-19 trajectories mandates to identify new therapeutics with potent SARS-CoV2 inhibitory activity. The repurposing of approved drugs is among the best and rapid strategies to identify potential therapeutics [9]. In this context, the computational techniques can help quickly identify novel molecules that target viral proteins to suggest candidates for repurposing. Hence, during the COVID-19 pandemic a lot of studies have been reported using a variety of such strategies [10], [11], [12]

The *in-silico* studies have helped in identification of many drugs that can target viral proteins viz. RNA-dependent RNA polymerase (RdRp), Spike, proteases (3CL^pro^ and PL^pro^) and human proteins such as angiotensin converting enzyme 2 (ACE2), transmembrane serine protease (TMPRSS2), and PIKfyve etc. [13]. Among them zanamivir, indinavir, saquinavir, and lopinavir are notable [14], [15]. There are many drugs such as baricitinib [16], favipiravir [17], duvelisib, ivermectin [18] and arbidol [19] etc., that are currently under clinical trials (https://clinicaltrials.gov/) to treat SARS-CoV-2 infection. In the early stage of the COVID-19 pandemic, remdesivir and hydroxycholoroquine were widely used for treatments of SARS-CoV2. Later on, it became evident that despite the efficacy observed *in-vitro*, hydroxycholoroquine did not show any clinically relevant benefits [20]. Recent reports suggests that the SARS-CoV2 not only causes infection in the lungs but may also cause infection in brain tissues [21]. The experiments on mice have shown that the SARS-CoV2 infections can cause neuronal distruction and death [22]. In COVID-19 also similar effects have been seen [23]. In light of the above, the therapeutic agents with good CNS penetration ability could have additional advantage [24].

Only a few studies have reported targeting more than one viral protein with a single molecule or using combination therapy [25], [26], [27], [28], [29]. In this study, we attempted to identify molecules that can simultaneously bind to multiple proteins of the SARS-CoV2. The strategy to target multiple proteins originates from the fact that individual viral proteins play specific role in multiple aspects of viral lifecycle such as attachment, entry, replication, morphogenesis and egress. Single molecules that can potentially target many viral proteins can perturb viral lifecycle at multiple points and thereby can be highly efficient in curbing SARS-CoV2 infection. Such strategy will also have a higher barrier towards emergence of resistant mutants.

In this work, we have used the 3D-structures of the SARS-CoV2 proteins to identify FDA approved drugs that can bind to these proteins using computational methods. The FDA approved drugs were chosen so that they can be quickly repurposed for treating COVID-19. Additionally, we also analyzed if the identified molecules can affect the host proteins that get differentially expressed as a result of SARS-CoV2 infection. We have also tested these molecules using an *in-vitro* SARS-CoV2 infection model in Vero E6 cells. These molecules can be used as modulators of both the SARS-CoV2 and human proteins.

## 2 Methods

### 2.1 Protein structure modelling

The SARS-CoV2 proteins for which there is no crystal structure reported were modelled using Modeller v9.22 [30] (homology modeling) (Table 1). The modelling template for each protein was identified by performing Delta-BLAST against the PDB database. Proteins were modelled using either single or multiple templates based on the query coverage. Further, the model stereochemistry and other structural parameters were assessed using standalone PROCHECK [31] tool.

**Table 1:** The details of the proteins selected for screening.

S. No.	Protein Name	Length*	RefSeq ID	Source$
1	Spike protein	1273	YP_009724390.1	PDB: 6VSB
2	Nucleocapsid phosphoprotein	419	YP_009724397.2	PDB:6WJI
3	PL^Pro^ (Nsp3)	1945	YP_009724389.1	PDB:6W02
4	Main protease (3CL^Pro^, Nsp5)	306	YP_009725301.1	PDB:6W63
5	RNA-dependent RNA polymerase (Nsp12)	932	YP_009725307.1	PDB:7BV2
6	Helicase (Nsp13)	601	YP_009725308.1	PDB:6XEZ
7	3′-to-5′ exonuclease (Nsp14)	527	YP_009725309.1	Homology modelled
8	EndoRNAse (Nsp15)	346	YP_009725310.1	PDB:6VWW
9	2′-O-ribose methyltransferase (Nsp16)	298	YP_009725311.1	PDB:6W4H

* Number of amino acids.

$ PDB/homology modelling.

### 2.2 Molecular docking of FDA approved drugs in SARS-CoV2 proteins

The ensemble docking approach increases the efficiency by allowing virtual screening against multiple conformations [32]. Therefore, the selected protein structures were subjected to 20 ns MD run (total 180 ns) using NAMD 2.6 [33] to explore the flexibility of the binding site (Supplementary methods Sec 1.1). The health of the molecular dynamics simulation was evaluated for stability using root mean square deviation (RMSD) (Supplementary Fig. 2), and radius of gyration (ROG) (Supplementary Fig. 3A-I). Thereafter, five snapshots were generated at equal time (4 ns) points during MD simulation for each protein. The structures of the FDA approved drugs were obtained from the e-drugs (https://chemoinfo.ipmc.cnrs.fr/TMP/tmp.13454/e-Drug3D_1993.sdf) repository containing 1993 molecules in the current library (updated till July 2020). The molecules were prepared by Schrödinger LigPrep wizard ligands using the default parameters. The protein structures were prepared by addition of missing atoms, hydrogens, assignment of bond orders and proper protonation states. The structure of each of the protein was minimized by keeping heavy atoms fixed and then the whole structure was minimized until a RMS gradient of 0.3 kcal/mol/Å as implied in Schrodinger.

The active site of the modelled proteins were identified using either of the following methods 1) the ligand binding pocket, if the co-crystal structure is available or 2) the ligand bound co-crystal structure of a close homolog or 3) the active site was predicted using SiteMap algorithm in Schrodinger v9.3 molecular modelling software [34]. The proteins with active site pocket volume of <150 Å^3^ were removed as smaller pockets may not be amenable to docking. The pockets were further selected by sequence comparisons and available literature. Finally, 9 proteins were selected for docking. The molecular docking was performed using the Glide module of Schrodinger molecular modelling software (www.schrodinger.com/glide). The docking was performed using default settings except that the formation of intramolecular hydrogen bonds was rewarded and the enhancement of planarity of conjugated π groups was checked on. Strain correction terms were applied, thus, if a docking pose has high internal strain, the docking score will be penalized and the pose may get removed from final results. A maximum of 10 poses were generated for each of the molecules. The final ranking of the molecules was obtained by calculating the average glide score in the five snapshots of a viral protein generated by molecular dynamics simulation to include the effect of binding site dynamics. The molecules showing a docking score of −8.5 [11] or better were selected for further analysis.

### 2.3 Binding free energy calculation (MM-GBSA):

The obtained hits were subjected to MM-GBSA analysis as implied in Glide module of Schrodinger modeling software for further selection of better hits. The receptor residues within 5 Å of the ligands were considered flexible for the MM-GBSA procedure with other default settings. Since the MM-GBSA binding energies reflect approximate free energies of binding, a more negative value indicates stronger binding. Similar to average glide score, average MMGBSA score was also calculated for each of the ligand for each viral protein.

### 2.4 The differential gene expression (DEGs) and protein–protein interaction network analysis

The differentially expressed genes were obtained from the data reported by Blanco-Melo et al. [35]. The identified DEGs were mapped for their interactions with other human proteins using HIPPIE v2.2 which contains 14,855 proteins and 411,430 interactions. The reported protein–protein interactions with a minimum score of 0.63 (medium confidence, 2nd quartile) [36] were used for creation of the network using Cytoscape v3.7.2. The largest interconnected component was extracted and degree for individual nodes was calculated to assess their importance in the network.

### 2.5 Interaction with human proteins

The drug-gene interaction database (DGIdb) that contains information about the drugs and their target genes was employed to identify the drugs that can modulate the differentially expressed genes in COVID-19. The drug gene interaction was obtained from various online resources such as Drugbank, and BindingDB. It was utilized to identify the drugs that can target both the viral as well as human proteins. Further, it was analysed whether a drug is agonist or antagonist for a given human protein for optimum therapeutic effect.

**Fig. 1:**
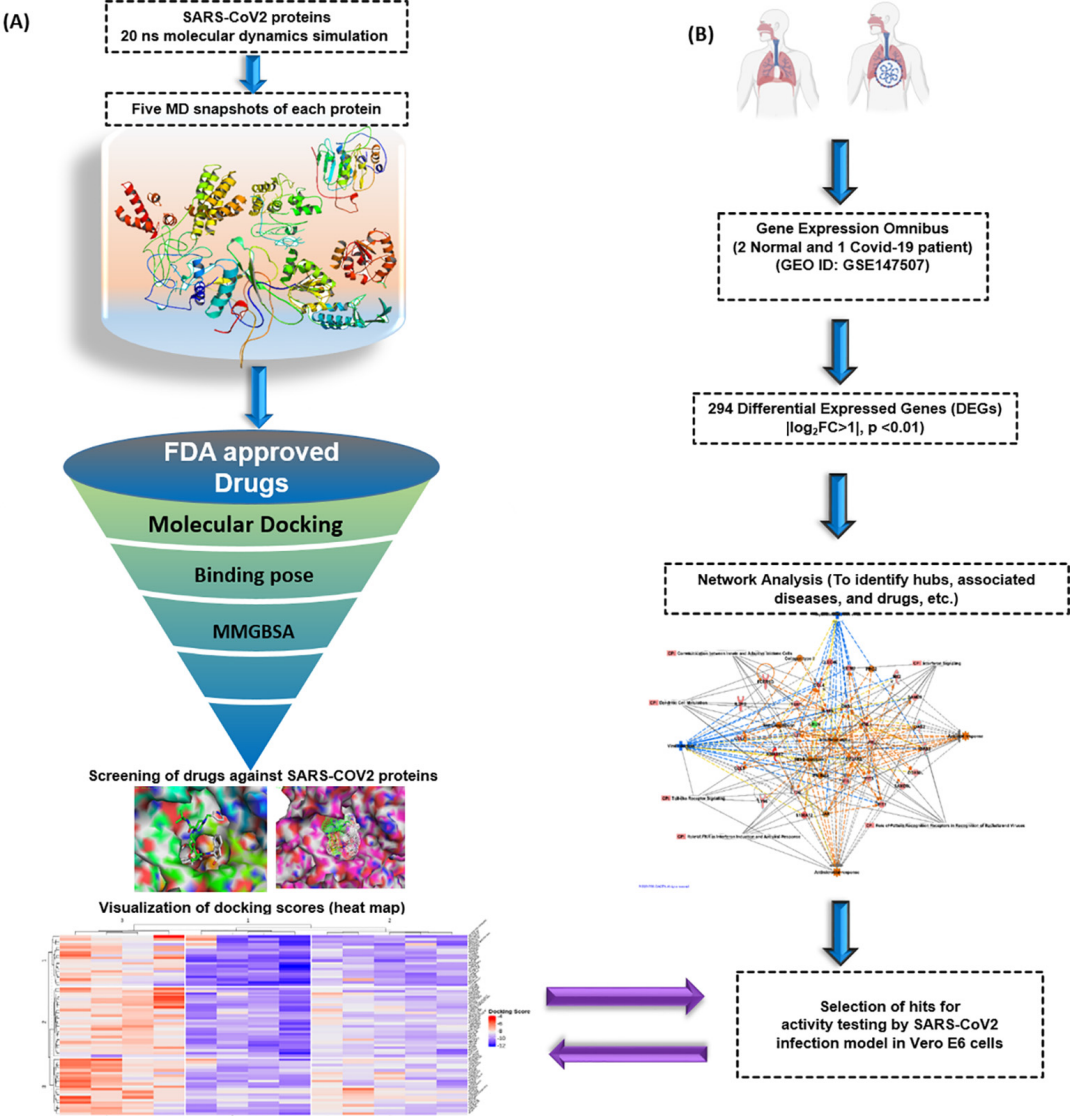
Outline of the repurposing work. (A) Nine SARS-CoV2 proteins structures were obtained from either PDB or modeled. The protein structures were subjected to 20 ns MD run (total 180 ns) to explore the flexibility of the binding site. Snapshots were generated at equal time points during MD simulation for each protein. The FDA approved drug library was docked in the generated snapshots using Glide. The MMGBSA score was also calculated for each of the ligand in individual snapshots. (B) Transcriptomics data from SARS-CoV2 infected and normal human samples identified significantly differentially expressed genes (|log_2_FC > 1|, p-value < 0.01) as a result of infection. A protein–protein-interaction network was created using these genes. They were also analyzed for their involvement in biological pathways using Ingenuity Pathways Analysis (IPA). The drug gene interactions were also analysed and molecules were tested using SARS-CoV2 infection model in Vero E6 cells for antiviral activity.

The calculations were performed on a high performance Linux cluster. The flowchart of the methodology is presented in Fig. 1.

*Virus and cells*: Vero E6 cells were obtained from the American Type Culture Collection (ATCC CRL-1586) and maintained at 37 °C with 5% CO2 in Dulbecco’s modified Eagle’s medium (DMEM; Gibco), supplemented with 10% heat-inactivated fetal bovine serum (Gibco) and 1× Pen-Strep solution (Gibco). SARS-CoV-2 virus (IND/ILS-01/2020, Genebank Accession MW559533) of clade 19A was isolated from the oropharyngeal swab sample of laboratory confirmed COVID-19 individual and was propagated in Vero E6 cells. Viral titers were determined by TCID_50_ assays in Vero cells. All experiments using SARS-CoV2 were performed in the Biosafety level 3 containment facility at the Institute of Life Sciences, Bhubaneswar. The approval for performing the drug repurposing studies against SARS-CoV2 was obtained from the institutional biosafety committee (No: V-122-MISC/2007-08/01).

*Drug Assay*: The FDA-approved drug library obtained from Enzo Life Sciences (BML-2843-0100, V.1.0) was used to source the compounds. 10 mM stocks were prepared in DMSO for further testing. To evaluate the effect of compounds in vitro, Vero E6 cells were seeded in 96 well culture plates. 16 h after seeding the cells were infected with SARS-CoV2 virus at 0.1 MOI for 2 h at 37 °C. The virus inoculum was replaced with fresh 2% FBS media containing the compounds at various dilutions. After 24 h incubation the cells and supernatants were collected to quantify cell-associated (intracellular) and cell-free (extracellular) viral loads by quantitative real-time RT-PCR using Takara PrimeScript™ One Step RT-PCR Kit (RR055A) with forward (5′-GTGAAATGGTCATGTGTGGCGG-3′) and reverse (5′-CAGATGTTAAA GACACTATTAGCATA-3′) primers and probe (5′-FAM-CAGGTGGAACCTCATCAG GAGATGC-BHQ-3′) targeting the SARS-CoV2 RdRp gene. Standard curve for absolute quantification of viral genome copies was generated using log-fold dilutions of plasmid pLVX-EF1alpha-nCoV2019-nsp12-2xStrep-IRES-Puro plasmid harbouring the SARS-CoV2 RdRp gene [37]. RT-PCR assay was performed on ABI 7500, Applied Biosystems PCR machine. Dose-response curve analysis (GraphPad Prism) was used to determine the half maximal inhibitory concentrations (IC_50_) of the compounds. Cytotoxicty of the compounds was evaluated in Vero E6 cells using the cell cell Vibryant MTT assay kit (Thermo Scientific).

*Immunofluorescence Assay*: Vero E6 cells were seeded in 96 well culture plates. 16 h after seeding the cells were infected with SARS-CoV2 virus at 0.1 MOI for 2 h at 37 °C. The virus inoculum was replaced with fresh 2% FBS media containing the compounds at their IC50 concentration. After 48 h incubation cells were washed with 1× PBS and fixed with 4% paraformaldehyde. Subsequently the cells were permeabilized and blocked for 1 hr with PBS containing 0.1% TritonX-100 and 3% BSA and probed with primary antibody specific for SARS-CoV2 nucleocapsid (Abgenex, cat. No. 11-2003) overnight at 4 °C. After 3 washes with PBS, the cells were probed with anti-rabbit secondary antibody tagged with Alexa Fluor 568 (Invitrogen, Carlsbad, CA), for 1 h at RT. The cells were counter stained with DAPI for 10 min to stain the nucleus. Images were captured under a 20x objective using an Olympus IX83 inverted fluorescence microscope. Images were quantified using ImageJ software (National Institutes of Health, Bethesda, Maryland, USA).

## 3 Results

As stated earlier nine viral proteins (Table 1) were selected for molecular docking.

The computational analysis of ligands binding to various proteins is a powerful method to quickly identify potential molecules for further studies. These methods have been successfully used in various studies [38], [39], [40]. In the first stage, the molecules were docked into the SARS-CoV2 protein snapshots obtained by molecular dynamics using Glide module of Schrodinger in standard precision (SP) mode. The molecules were then ranked using average Glide score. The MM-GBSA was then performed to ensure the appropriate selection of top hits. All hits were visually inspected for interactions with receptor residues.

The following strategy was adopted for the current study to identify candidate drugs: 1. drugs that can inhibit viral entry into host cell by perturbing the function of surface glycoproteins like the spike. 2. blocking the functions of viral enzymes that plays a vital role in replication such as 2′-O-ribose methyl transferase, RNA-dependent RNA polymerase, endoRNAse, helicase, 3′-to-5′ exonuclease, 3C-like main protease and papain-like protease. 3. drugs that can also affect differentially expressed host proteins in COVID-19 along with the viral proteins.

### 3.1 Molecules docking to SARS-CoV2 structural proteins

The hallmark feature of coronaviruses is their transmembrane spike (S) glycoprotein as this protein is the reason for its name “Corona” in Latin meaning, “Crown”. SARS-CoV-2 uses its spike (S) protein to attach to host cells. The spike protein exists as homo-trimers. Each monomer is about 180 kDa and has two distinct subunits S1 and S2. While the receptor binding is mediated by S1 subunit with the help of receptor binding domain (RBD), the fusion between the viral envelope and the host cellular membranes is facilitated by the S2 subunits upon the cleavage of S1-S2 junction by host proteases [41]. The S1 subunit of spike protein in SARS-CoV2 has four distinct domains: NTD, CTD1, CTD2 and CTD3, of these the “up” conformation of CTD1 is responsible for binding with ACE2 receptor [42]. The S protein, due to its important role in the first stage of infection, is an important target for development of therapeutics and vaccines. The co-crystal structure of the S-protein with small molecule ligand is not available, therefore we used the sitemap algorithm in Schrodinger to identify the active site on S-protein. The sitemap revealed a site that is very close to the receptor binding domain and trimerization interface lined by the residues Ser 46, Leu 48, Leu 303, Lys 304, Ser 305, Glu 309, Thr 732, Asn 758, Thr 827, Phe 833, Tyr 837, Arg 847, Lys 854, Asn 856, Val 860, Gln 949, Val 952, Asn 955, Gln 957, Asn 960, Val 963, and His 1058. Many of these residues are highly conserved among coronaviruses. The site is overlapping to the site suggested by Kalathiya et al. [43]. Recently, some of the SARS-CoV2 strains containing mutants of the S-protein (D614G) with high infectivity have been reported. This mutant does not change the structure of S-protein but increases its binding with human TMPRSS2 protein [44]. This mutation is away from the identified binding site.

**Fig. 2:**
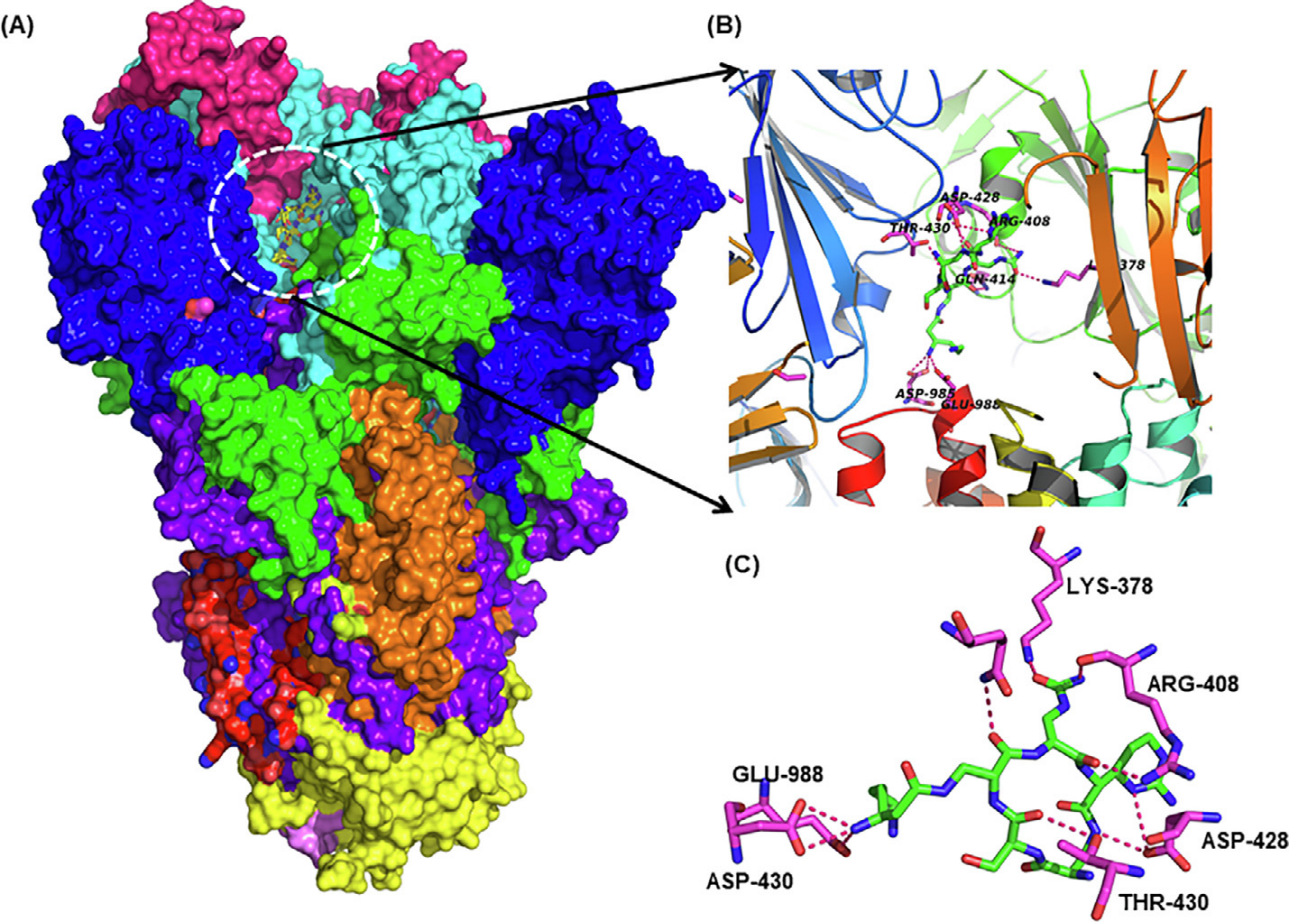
Virtual screening of FDA drugs against Spike protein. (A) The surface view of spike protein where domains are represented in different colors. The ligand binding site is shown inside white dotted circle at RBD (receptor binding domain) of the spike protein. (B) Cartoon depiction of the RBD showing the secondary structure elements and binding of capreomycin. (C) Closeup of RBD-capreomycin (average docking score −9.10) interaction showing residues making hydrogen bonds interactions (red dotted lines) with capreomycin. (For interpretation of the references to colour in this figure legend, the reader is referred to the web version of this article.)

Our molecular docking analysis suggest that capreomycin, posaconazole, mefloquine, nebivolol, angiotensin II, celecoxib and trimethoprim bind to spike protein with appreciable affinity (Supplementary Table 1) (Fig. 2). Other groups have also predicted the binding of posaconazole to spike protein which further substantiates our analysis [45]. Posaconazole is an antifungal agent used in the prevention of invasive fungal infections and is also shown to inhibit the entry of Chikungunya virus [46] and replication of Zika and Dengue viruses by binding to oxysterol-binding protein (sterol transporter) [47]. Mefloquine is an antimalarial drug used in chloroquine resistant malaria. Nebivolol is an antihypertensive molecule with a very good safety profile in subjects with obstructive respiratory comorbidities [48] and can be an important drug to consider in SARS like diseases. Capreomycin is a polypeptide (isolated from *Streptomyces capreolus*) used in the treatment of multidrug resistant tuberculosis. Its mechanism is similar to aminoglycosides and used in the inhalation therapy of pulmonary tuberculosis by spray-drying technology [49], [50]. It can be a promising prophylactic agent against SARS-CoV2 using similar application strategy (Fig. 2).

**Fig. 3:**
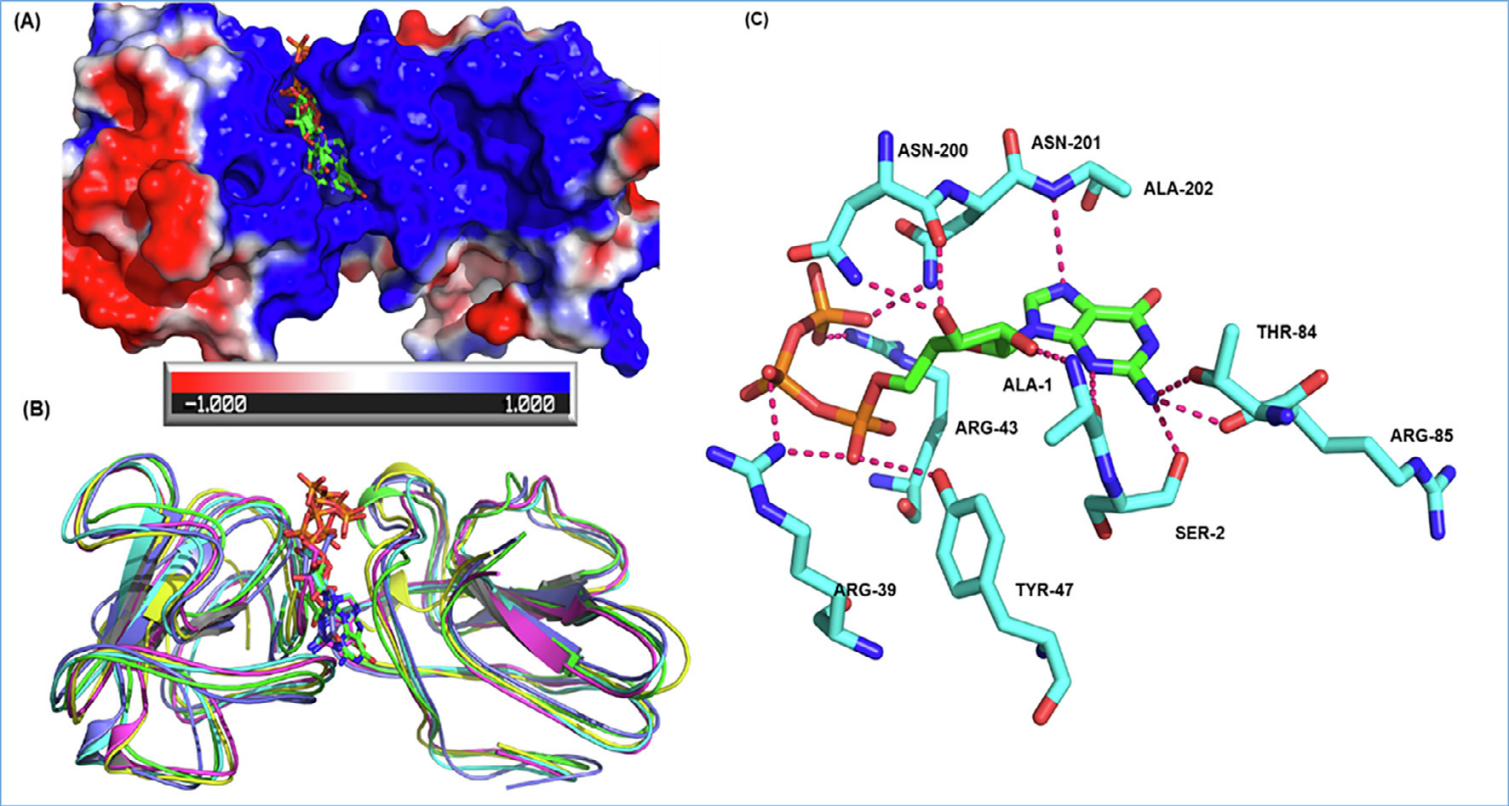
Nelarabine binding to nucleocapsid (N) protein of SARS-CoV2. (A) The surface is coloured by the charge on the amino acids. The red, white and blue surface area depict negative, neutral and positive surface respectively. Nelarabine binds in a predominantly positive area at the nucleocapsid homodimer interface. (B) Nelarabine docked in five MD snapshots of N-protein (average docking score −8.73). (C) Ligand-protein interaction showing nelarabine makes multiple hydrogen bond interactions with N-protein. (For interpretation of the references to colour in this figure legend, the reader is referred to the web version of this article.)

The nucleocapsid (N) protein is crucial for the viral RNA packaging. It is made up of two distinct RNA-binding domains (the N-terminal and the C-terminal domain) linked by serine/arginine-rich (SR-rich) domain (SRD)[51]. Previous studies with SARS-CoV1 suggest that N protein inhibits TGF-beta, AP-1, NF-kB signaling and type 1 interferon production but induces apoptosis. The sera of COVID-19 patients shows the presence of IgG, IgA, and IgM antibodies against N protein suggesting its role in eliciting humoral immune response [52], [53]. In the current study the crystal structure of N-terminal dimerization domain of nucleocapsid phosphoprotein with a ligand (PDB ID: 6WKP) is used. The ligand binds at the dimer interface and has interactions with residues of both of the chains. The active site was defined as residues lying within 5 Å of the cocrystallized ligand. Our study predicts that nelarabine, paclitaxel, regadenoson, quinaprilat and bromfenac are among top molecules binding to N protein (Supplementary Table 1) (Fig. 3).

### 3.2 Molecules binding to SARS-CoV2 enzymes:

2′-O-Methyl Transferase (Nsp16) of SARS-CoV2 belongs to the S-adenosylmethionine-dependent methyl transferase family and is activated upon binding to Nsp10. Nsp10 binds with a conserved four amino acid sequence ‘KDKE’ of Nsp16 in its catalytic pocket and activates its methyltransferase activity. Capping of viral mRNA at 5′-end is one of the viral strategy for protecting viral transcripts from host 5′ exoribonucleases and escaping the host innate immune response by mimicking as host mRNAs, thus Nsp16 is the potential target for antiviral therapeutics [54].

**Fig. 4:**
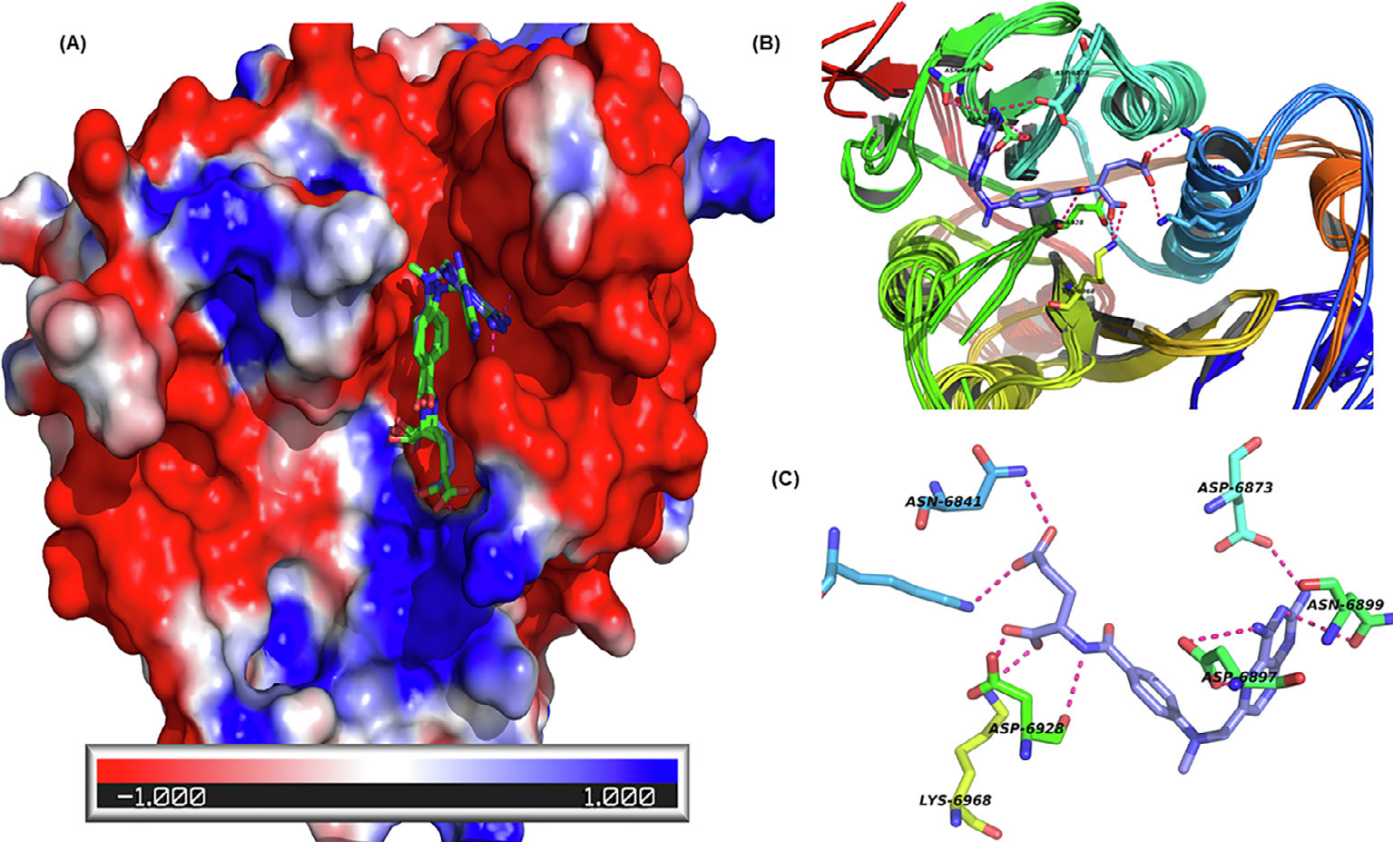
Methotrexate binding with methyltransferase (NSP16). (A) Representation of the NSP16 surface coloured by electrostatic charges on amino acids. The red, white and blue surface area depict negative, neutral and positive surface respectively. Methotrexate bind with methyltransferase in a predominantly negative cavity (average docking score −9.07). (B) The cartoon representation shows active site flexibility during MD simulation. (C) Ligands interaction plot shows methotrexate (purple sticks) forms hydrogen bonds with Asn6841, Asp6928, Lys6968, Asp6897, Asn6899 and Asp6876 of NSP16. (For interpretation of the references to colour in this figure legend, the reader is referred to the web version of this article.)

The crystal structure of SARS-CoV2 Nsp16 (PDB ID: 6W4H, co-crystallized with S-adenosyl methionine) was used in the current studies. The binding site was found to be lined by the residues Phe 70, Gly 71, Ala 72, Gly 73, Asp 99, Leu 100, Leu 111, Gly 113, Met 131, Tyr 132, Asp 133, Phe 149, Asp 114, Ala 116, Cys 115, and Val 118. Our study shows that methotrexate, viomycin, saralin, saquinavir, venetoclax, vidarabine, histrelin, triptorelin and ribavirin binds to Nsp16 with high affinity (Supplementary Table 1). Methotrexate forms hydrogen bonds with Asn6841, Asp6928, Lys6968, Asp6897, Asn6899 and Asp6876 of NSP16. (Fig. 4).

Methotrexate acts as an antimetabolite and thus used as an antineoplastic drug. It is also used in treatment of inflammatory diseases like rheumatoid arthritis. It decreases the de novo synthesis of purines and pyrimidines and forms dimers with thymidylate synthase (TS), hence also has anti-parasitic effect [55]. Methotrexate is also shown to effectively inhibit Zika and Dengue virus replication [56]. Zidovudine is used in HIV1 treatment (21), histrelin and triptorelin are gonadotropin-releasing hormone analogs used in the treatment of central precocious puberty and endometriosis [57]. Lanreotide is a long-acting analog of somatostatin and is used for the management of acromegaly, a condition caused by excess secretion of growth hormone. Octreotide is also a somatostatin analog currently used for the treatment of watery diarrhoea and flushes caused by certain carcinoid tumors. Vidarabine (ara-A) is a purine analog and an antiviral drug used for infections caused by herpes simplex and varicella zoster viruses.

Among all the proteins encoded by SARS-CoV2 genome, PL^pro^ (papain-like protease) and 3CL^pro^ (3C chymotrypsin‐like protease) are two important viral proteases that cleave the two polyproteins (pp1a and pp1ab) into individual viral proteins (Nsp2-Nsp16). The two proteases are important for replication and controlling the host cell response and hence they are among the key targets for the development of therapeutics against SARS-CoV2. These proteases have cysteine in the active site that has also been targeted for the development of covalent inhibitors. There are many small molecules, peptides and peptidomimetics that have been developed against these proteases [58], [59], [60].

**Fig. 5:**
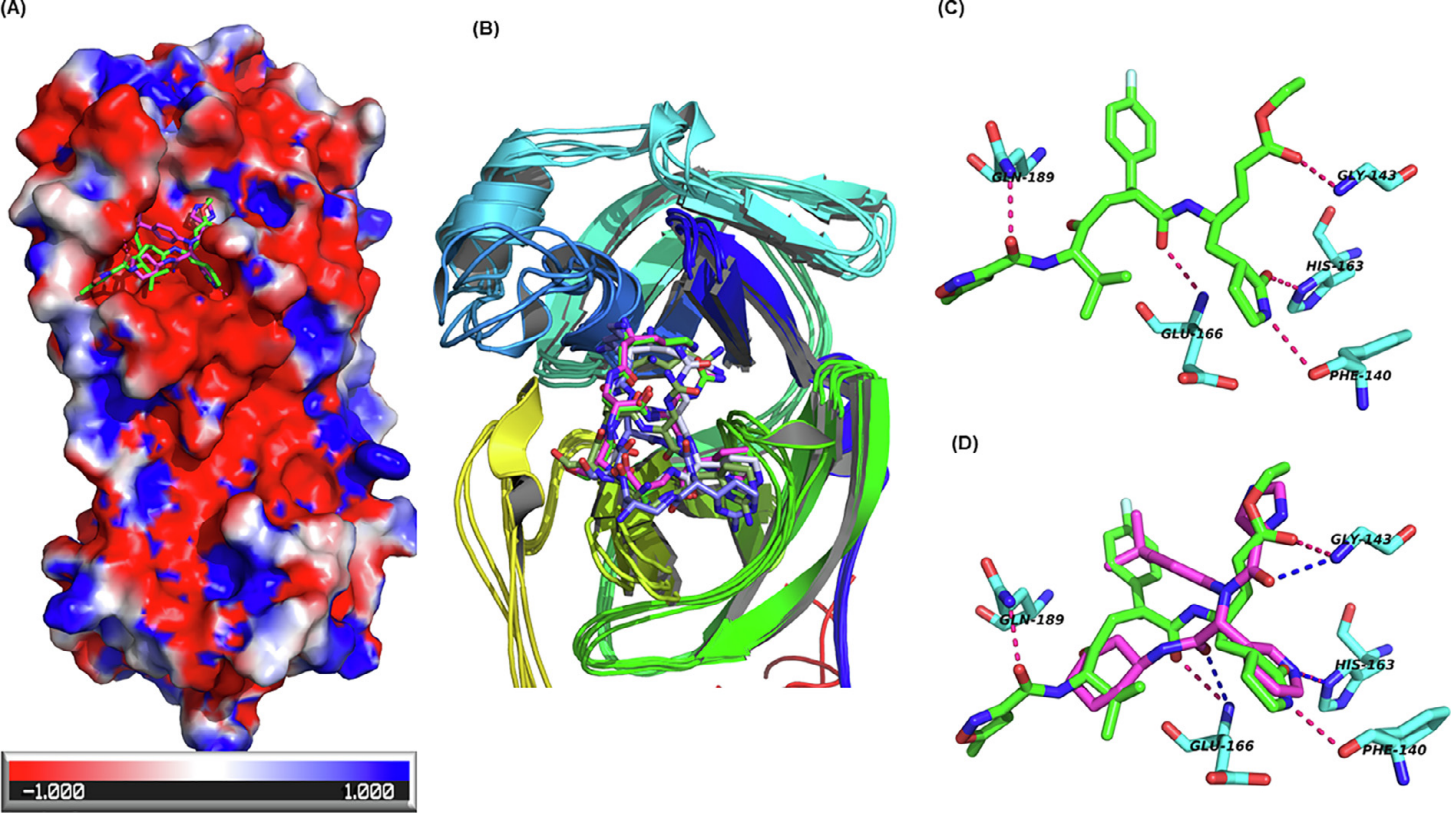
Binding of rupintrivir with the main protease of SARS-CoV2. (A) The red white and blue surface area depict negative, neutral and positive charged surface respectively on the main protease. Rupintrivir is shown in green sticks while the co-crystallized ligand is shown in maroon. (B) The docking of rupintrivir in MD snapshots of the receptor (average docking score −10.58). (C) The hydrogen bonding interactions (dotted lines) between rupintrivir (green sticks) and active site residues of main protease (N-(4-*tert*-butylphenyl)-N-[(1R)-2-(cyclohexylamino)-2-oxo-1-(pyridin-3-yl)ethyl]-1H-imidazole-4-carboxamide, cyan sticks). (D) Binding pose comparison of rupintrivir (green sticks) with co-crystallized ligand (maroon sticks). The receptor residues are shown in cyan sticks. The dotted line shows hydrogen bond interaction with the receptor. (For interpretation of the references to colour in this figure legend, the reader is referred to the web version of this article.)

The 3CL^pro^ is a cysteine protease having three domains: β-barrel Domain I (residues 8–101) and II (residues 102–184) and α-helix domain III (residues 201–306) similar in structure to chymotrypsin [61]. The functional protease is a dimer that cleaves polyprotein 1ab in 11 regions at its specific cleavage site (P1) of Leu-Gln↓(Ser, Ala, Gly). The sequences of SARS-CoV2 and SARS-CoV main protease are highly similar (96% identity) and so their 3D structures, barring some surface residues. However, enigmatically the inhibitors of SARS-CoV 3CL^pro^ lopinavir and ritonavir that were also recommended for use against SARS-CoV2 have not shown expected results in the clinical trials for COVID-19 [62]. The binding site for 3CL^pro^ was defined as residues falling within 5 Å of the co-crystallized ligand (PDB: 6 W63). The residues Thr 25, His 41, Cys 44, Thr 45, Ser 46, Met 49, Asn 142, Gly 143, Ser 144, Cys 145, His 164, Met 165, Glu 166, Leu 167, Pro 168, Asp 187, Arg 188, Gln 189, and Gln 192 were used for defining the active site. Rupintrivir, alatrofloxacin, cangrelor, capreomycin, naldemedine, lopinavir and indinavir are among the drugs predicted to bind to 3CL^pro^ (Supplementary Table 1) (Fig. 5). It is important to note that most of the molecules are making HB interactions with the oxyanion hole residues (Asn 142, Gly 143, Ser 144) of the 3CL^pro^.

Previous studies report α-ketoamides, lopinavir and ritonavir as inhibitor of 3CL^pro^ [63], [64]. Ruprintrivir inhibits human rhinovirus (HRV) 3C protease and has shown broad-spectrum anti-HRV activity [65]. Others have also indicated it to be useful against SARS-CoV2 main protease [66]. Indinavir is shown to inhibit HIV protease by blocking its active site and leads to immature virus particle formation, however high doses have been linked to lipodystrophy syndrome [67]. Naldemedine, is a μ-opioid receptor antagonist used for the treatment of opioid-induced constipation [68].

**Fig. 6:**
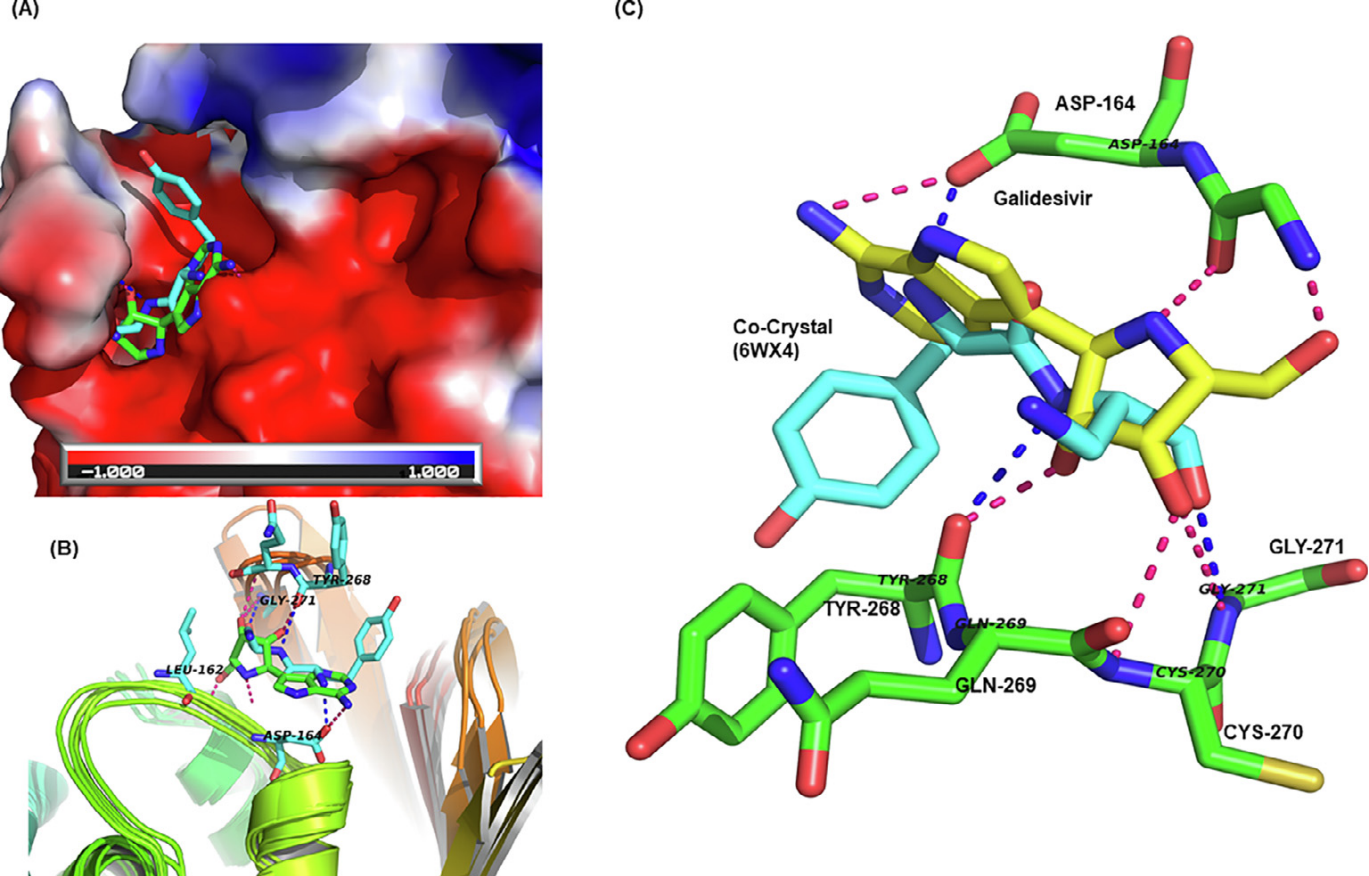
Binding of galidesivir with PL^pro^ (NSP3)**.** (A) The surface is colored as a function of electrostatic charge on the residues. The co-crystallized ligand (cyan sticks) and galidesivir (green sticks) (average docking score −11.90). (B) Superimposition of the snapshots and binding of galidesivir. (C) A close up of galidesivir-PL^pro^ interactions. Hydrogen bonding interactions are shown for galidesivir (yellow sticks) and co-crystallized ligand (ADENOSINE-5-DIPHOSPHORIBOSE, cyan). The pink and blue dotted lines show hydrogen bonds galidesivir and co-crystallized ligand respectively. (For interpretation of the references to colour in this figure legend, the reader is referred to the web version of this article.)

PL^pro^ is a domain within nsp3 of pp1a/pp1ab with proteolytic activity. It cleaves three sites at 181–182, 818–819, and 2763–2764 at the N-terminus of PP1ab [69]. It is the least explored among coronavirus proteins and only a few inhibitors are known for this protein [70]. Our study predicts that galidesivir, pralatrexte, methotrexate, daunorubicin, ganciclovir, folic acid, montelukast and itraconazole are among the molecules binding to the protease PL^pro^ (Supplementary Table 1). Galidesivir has broad-spectrum antiviral activity (in vitro) against many RNA viruses in nine different families, including the coronaviruses [71]. The binding of galidesivir with PL^pro^ is shown in Fig. 6. This drug has been under clinical trials for COVID-19 (NCT03891420). Daunorubicin (DNR) is the anthracycline compound used in the Kaposi's sarcoma and lymphoma treatment of HIV-1 infected patients [72]. Moreover its derivative N,N-dimethyl daunomycin (NDMD) is used as the inhibitor of Herpes simplex virus (HSV) [73]. Montelukast has been predicted by other groups as well to bind to main protease of SARS-CoV2 [74]. An interesting observation is the identification of folic acid as a high affinity ligand of PL^pro^.

**Fig. 7:**
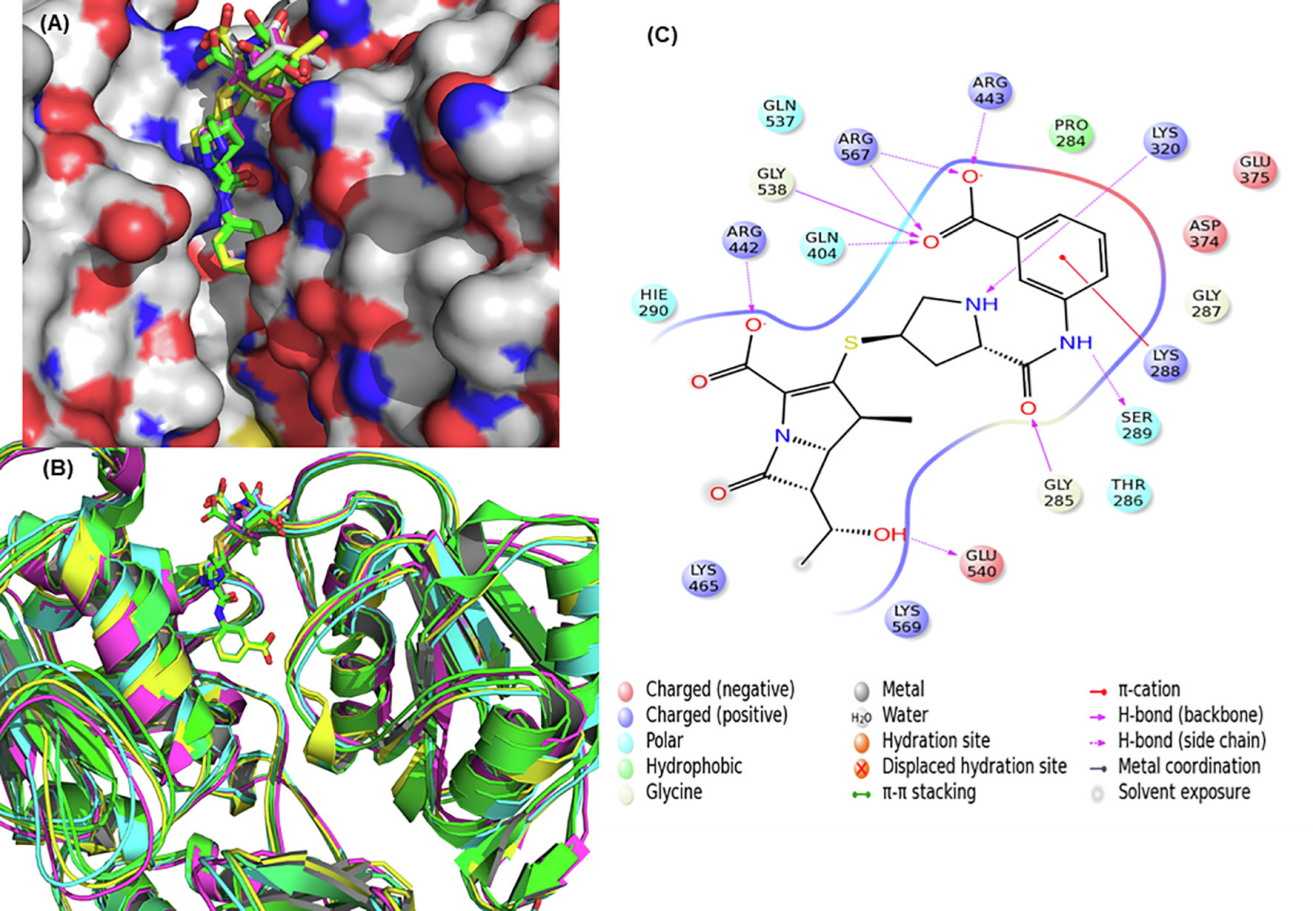
(A) Binding of eratapenem (average docking score −9.86) at the helicase protein of SARS-CoV2. (B) The five receptor frames are shown with docked eratapenem. (C) The ligand plot is showing interactions between eratapenem and helicase binding site.

Helicase enzyme (Nsp13) of SARS-CoV2 is motor protein essential for unwinding of both dsDNA and dsRNA and has metal binding (Zn2 +) N-terminal and helicase domain (Hel). It is involved in formation of RTC of SARS-CoV2 along with RdRp, which is known to enhance its activity [75]. The SARS-CoV2 helicase has 99.8% sequence similarity with that of SARS-CoV. Since it is one of the most conserved proteins in Nidoviruses and is essential for viral RNA synthesis, it is an attractive target for antiviral drug development. A recent review summarizes its importance as a drug target in COVID-19 [76]. In the current studies the cryo electron microscope structure of helicase-RdRp (PDB: 6XEZ) was used. The residues within 5 Å of the ADP bound to helicase enzyme were defined as the active site. Our analysis shows that eratapenem, methotrexate, clofarabine, trimethoprim, ascorbic acid, cefixime, and pibrentasvir bind to the helicase with high affinity (Fig. 7). Clofarabine is a potent HIV-1 inhibitor [77]. Pibrentasvir, is a HCV NS5A inhibitor effective against all HCV genotypes [78].

The most vital enzyme responsible for the replication/transcription of the viral genome is the RNA-dependent RNA polymerase (RdRp) also known as Nsp12. The primer for RdRp RNA synthesis is synthesized by Nsp8 [79]. Nsp12 has two main functional domains namely nidovirus RdRp associated nucleotidyl transferase (NIRAN) domain and RNA dependent RNA polymerase (RdRp) domain. The NIRAN domain helps in nucleotide transfer while RdRp domain is involved in the polymerisation. The RdRp is conserved in structure and function among RNA viruses [80]. This enzyme, due to its importance in viral replication and also to the fact that humans are devoid of it, is a very attractive target [81], [82]. Moreoever due to the availability of its structure with cofactors Nsp7 and Nsp8 (PDB: 6 M71) and remdesivir (PDB: 7BV2) the structure based design is feasible. A number of studies have been done on development of RdRp inhibitors and some molecules e.g. remdesivir, favipiravir etc. have been approved for emergency use in COVID-19.

**Fig. 8:**
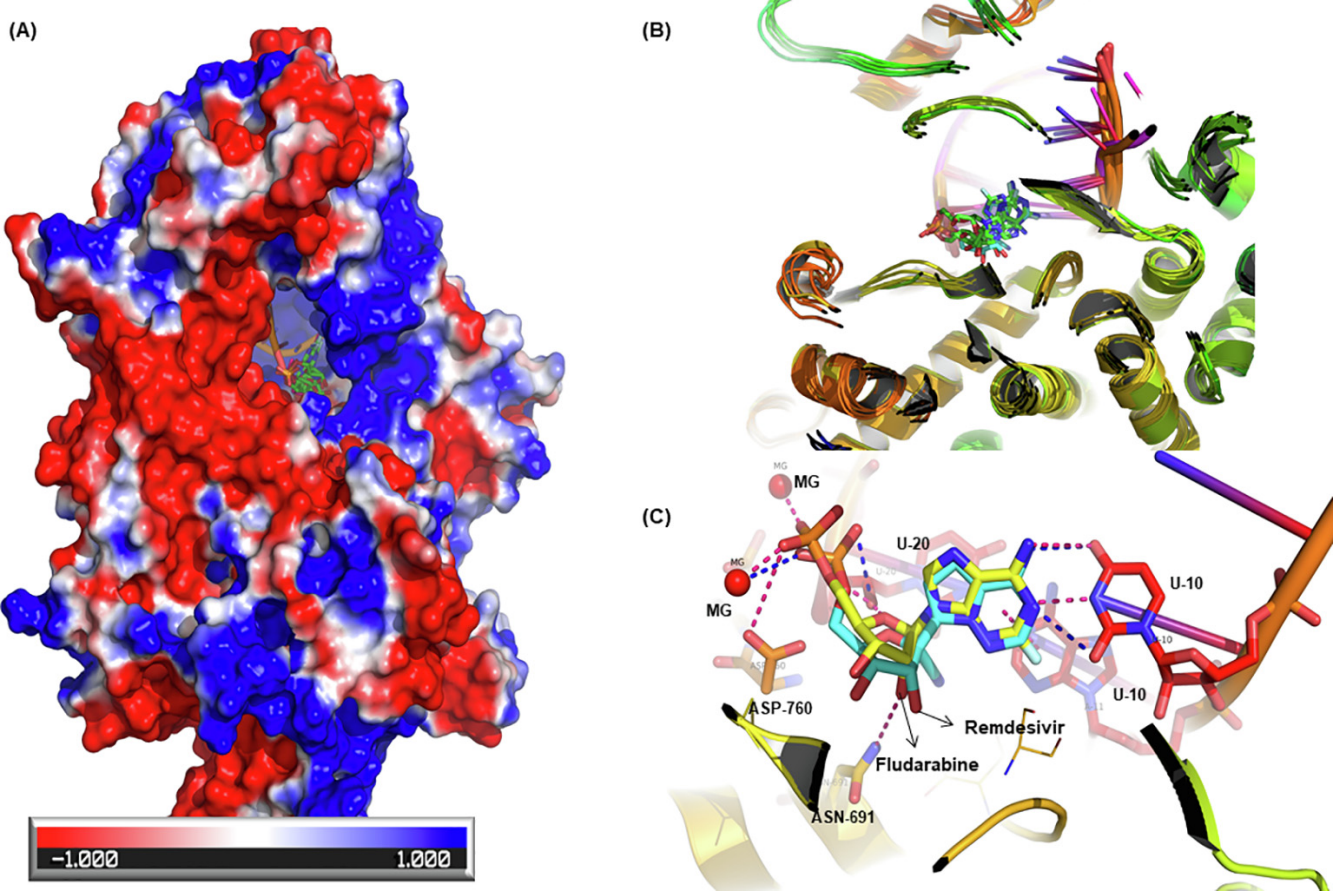
Binding of fludarabine with RNA-dependent RNA polymerase (RdRp) of SARS-CoV2. (A) The surface view of RdRp protein with co-crystallized ligand (remdesivir). The red white and blue surface area depict negative, neutral and positive charged surface respectively. (B) Fludarabine docked into MD snapshots of RdRp (average docking score −11.04). (C) Fludarabine (yellow sticks) and remdesivir (blue sticks) in RdRp active site. The hydrogen bonding interactions are shown in dotted lines (blue-remdesivir, red-fludarabine). It is clear from the binding pose and interactions that fludarabine binds to RdRp quite similar as remdesivir. (For interpretation of the references to colour in this figure legend, the reader is referred to the web version of this article.)

In the current studies, we have used the structure of RdRp complexed with remdesivir (PDB: 7BV2). The residues falling withing 5 Å of the remdesivir were defined as active site. Our analysis shows that fludarabine, cobicistat, capreomycin, regadenoson, doxazocin, pibrentasvir, elbasvir, indinavir and remdesivir among others that can bind with RdRp (Fig. 8).

Fludarabine is used for the treatment of hematological malignancies. It inhibits various critical enzymes and results in the inhibition of DNA synthesis. It has been predicted to be active against SARS-CoV2 RdRp by other groups as well [74], [83]. Ribavirin is broad spectrum antiviral used for treatment of RSV infection, hepatitis C and viral hemorrhagic fevers [84]. It is a well known RdRp inhibitor. Cobicistat is known to inhibit the cytochrome-mediated metabolism of HIV protease and was approved in 2012 by FDA as pharmacoenhancer for HIV treatment [85]. Other groups have also predicted that cobicistat and capreomycin can inhibit SARS-CoV2 protease [86], [87]. Pibrentasvir and elbasvir are HCV NS5A inhibitors and indinavir is potent HIV protease inhibitor [88]. Another molecule monteleukast, a leukotrine inhibitor used as antihistamine was also showing good affinity towards RdRp (docking score −9.42). The molecules we identified to bind to RdRp can serve as potential alternatives to remdesivir.

**Fig. 9:**
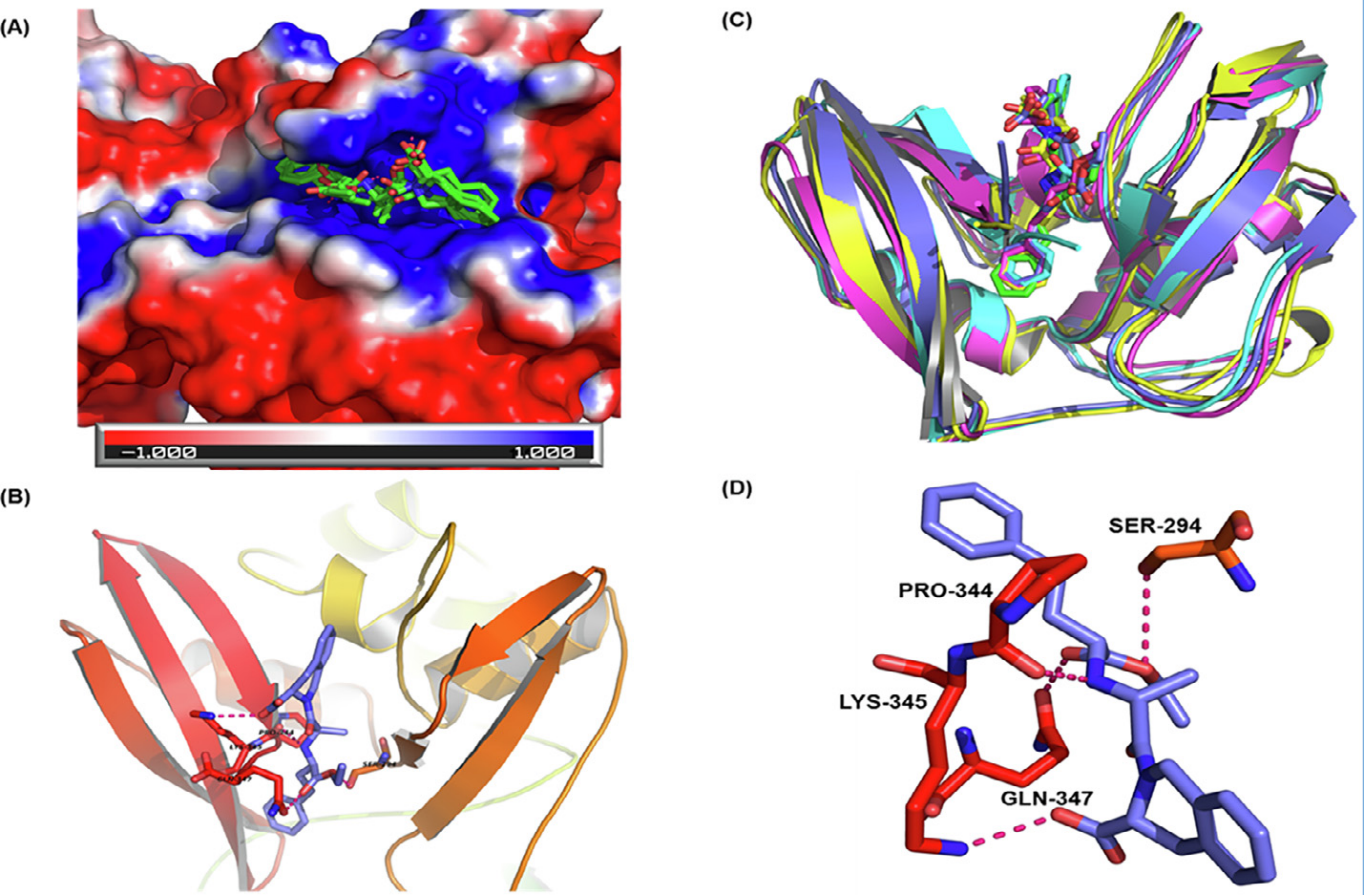
Quinapril binding to endonuclease protein (Nsp15): (A) The surface view of endonuclease with docked ligands. The red white and blue surface area depict negative,neutral and positive charged surface respectively. Ligands bind to the predominantly positive cavity. (B) The MD frames with ligand (average docking score −10.02). (C) The secondary structural elements of the binding site (cyan) and quinapril (purple). (D) Hydrogen bonding interactions between quinapril (purple) and receptor residues Gln347, Lys345, and Ser294 (red). (For interpretation of the references to colour in this figure legend, the reader is referred to the web version of this article.)

The Nsp15 is EndoRNase with endoribonuclease activity. It cleaves the 5′ and 3′ of uridylate residues in RNA by forming 2′-3′cyclic phosphodiester. Its mechanism is similar to that of RNase A, RNAse T1 and XendoU [89]. Its NendoU activity can interfere with the host’s innate immune response and masks the exposure of viral dsRNA to host dsRNA sensors [90]. The crystal structure of SARS-CoV2 Nsp15 cocrystallized with U5P (PDB: 6WLC) was used in the current studies. The active site was defined by the residues falling with 5 Å of the co-crystallized ligand. The active site is situated near the N-terminal and is surrounded by beta sheets and a helix. In our analysis, drugs such as quinapril, octreotide, folic acid, and macimorelin were found to bind to Nsp15 with appreciable affinity (Fig. 9). Quinapril is an angiotensin converting enzyme (ACE) inhibitor and the ACE inhibitors have been suggested to be beneficial for COVID-19 patients [91]. Folic acid is essential for DNA and protein synthesis and in the adaptive immune response [92]. The dose dependent effect of folic acid on rotavirus infected mice has been reported indicting its antiviral activity [93]. Additionally, the role of folic acid in the prevention of cellular entry of SARS-CoV2 has been reported [94]. Macimorelin is used for the diagnosis of adult growth hormone deficiency [95]. Interestingly other groups have also predicted it to be active against SARS-CoV2 [96], [97].

Nsp14 is the 3′-5′exonuclease that plays a role in proofreading mechanism [98]. Nsp14 contains four conserved DE-D-D acidic and a zinc-finger (ZnF) domain [99]. The homology model of Nsp14 based on the crystal structure of closely related Nsp14 of SARS CoV (PDB: 5C8T_chainB, 95.07% identity) was used for the current studies. The binding site was defined by comparison with the cocrystallized ligand (PDB: 5C8T, chainB). The SARS-CoV2 Nsp14 active site was found to be lined by the residues Arg289, Trp292, Asn306, Arg310, Asp324, Lys336, Asp362, Ala363, Leu366, Asn386, Asn388, Phe401, Tyr419, Asn422, Phe426, His455, Arg476, Tyr491, and Phe506.

Our molecular docking predicted that cangrelor, venetoclax, pimozide, nilotinib, droperidol, nebivolol, indacaterol, ezetimibe, simeprevir, siponimod, lapatinib, elagolix bind to Nsp14 (Supplementary Fig. 1).

Pimozide, a calmodulin inhibitor is shown to inhibit Chikungunya virus secretion [100]. Moreover, it binds to the envelope protein of HCV and inhibits infection with many HCV genotypes [101]. Droperidol is also predicted by other groups to be effective against SARS-CoV2 infection [102]. Ezetimibe is shown to inhibit formation of capsid-associated relaxed circular DNA of Hepatitis B Virus (HBV) [103] and is also shown to inhibit Dengue infection by interfering in formation of replication complex [104]. Indacaterol is the β2-adrenoceptor agonist and used in the treatment of chronic obstructive pulmonary disease (COPD) since it induces bronchodilation [105]. It is a promising candidate for therapeutics against SARS-CoV2 due to its ability to regulate genes involved in suppressing proinflammatory cytokine production and attenuation of airway hyper-responsiveness [106]. However, dose and treatment schedule needs to be evaluated due to its counter effect on the expression of RNase L which is vital for antiviral response.

Since one of our major objectives was modeling of the intrinsic flexibility of the SARS-CoV2 proteins by molecular dynamics simulation and finding drugs that can adjust with the site flexibility. We provide a summary of the top drugs for individual proteins and their docking scores in the frames generated by molecular dynamics along with the average MMGBSA score Table 2. The drugs with consistently good docking scores will have a better average. This approach is novel and is not reported anywhere before for screening of drugs against SARS-CoV2 as per the best of our knowledge.

**Table 2:** Docking and MMGBSA scores of top drugs targeting different SARS-CoV2 proteins.

Drugs/Frames	F1$	F2$	F3$	F4$	F5$	Avg_score*	MMGBSA_Avg#
RdRp							
Fludarabine	−11.32	−10.85	−9.83	−11.34	−11.86	−11.04	−126.47
Ribavirin	−10.83	−9.63	−10.24	−10.06	−11.38	−10.43	−110.14
Ivermectin	−10.94	−10.28	−9.08	−9.28	−10.04	−10.30	−99.25
Remdesivir	−10.53	−9.69	−8.49	−10.02	−10.21	−9.79	−107.23
Cangrelor	−9.39	−8.36	−9.03	−9.42	−10.36	−9.31	−118.86
Nebivolol	−9.56	−9.89	−8.69	−7.98	−10.13	−9.25	−88.87

**Spike**							
Capreomycin	−9.61	−8.82	−9.16	−8.24	−9.68	−9.10	−168.25
Hydroxychloroquine	−9.39	−9.02	−8.97	−8.14	−9.26	−8.96	−85.29
Mefloquine	−8.56	−8.69	−7.89	−9.02	−9.10	−8.65	−105.84
Nebivolol	−8.56	−8.59	−8.47	−7.89	−9.26	−8.55	−135.63
Angiotensin II	−10.77	−8.68	−7.28	−8.02	−7.89	−8.53	−96.82
Celecoxib	−9.23	−7.58	−8.08	−7.89	−8.95	−8.35	−83.21

**Main Protease (3CL^pro^)**							
Mefloquine	−10.08	−9.88	−10.03	−10.09	−10.25	−10.06	−78.23
Rupintrivir	−10.68	−10.56	−9.87	−10.78	−11.03	−10.58	−110.61
Lopinavir	−11.04	−9.58	−9.97	−10.89	−11.31	−10.56	−92.06
Lapatinib	−10.03	−10.56	−10.00	−10.97	−11.03	−10.52	−87.03
Ritonavir	−10.77	−10.26	−10.36	−10.02	−10.98	−10.48	−85.06
Fosaprepitant	−9.89	−9.85	−10.24	−10.36	−10.58	−10.18	−93.05

**Exonuclease**							
Cangrelor	−11.79	−10.58	−11.06	−11.03	−11.28	−11.15	−147.54
Venetoclax	−9.90	−10.03	−10.07	−9.58	−10.01	−9.92	−165.58
Pimozide	−9.68	−9.50	−9.89	−10.05	−10.21	−9.87	−90.28
Nebivolol	−9.23	−9.45	−10.25	−10.84	−9.58	−9.87	−115.42
Nilotinib	−9.46	−9.65	−9.89	−10.58	−9.25	−9.77	−105.87
Droperidol	−9.34	−9.26	−10.21	−9.87	−8.51	−9.44	−100.52

**EndoNuclease**							
Octreotide	−9.40	−8.12	−8.58	−10.87	−11.03	−9.60	−86.25
Quinapril	−9.63	−8.69	−9.02	−9.36	−10.02	−9.34	−108.02
Celecoxib	−9.47	−9.57	−8.24	−10.24	−9.02	−9.31	−98.57
Ribavirin	−8.06	−9.28	−8.26	−10.03	−10.58	−9.24	111.58
Folic Acid	−8.89	−8.98	−9.36	−8.14	−10.28	−9.13	−99.58
	−8.52	−7.85	−9.28	−8.97	−10.03	−8.93	−108.20

**Helicase**							
Eratapenem	−10.34	−9.51	−9.67	−10.21	−9.58	−9.86	−120.58
Methotrexate	−9.99	−8.28	−10.69	−9.14	−10.78	−9.78	−111.21
Trimethoprim	−8.25	−8.59	−9.41	−9.26	−9.57	−9.02	−105.13
Ivermectine	−9.07	−8.82	−9.26	−8.87	−9.05	−8.99	−98.82
Clofarabine	−8.33	−8.55	−8.69	−8.78	−9.87	−8.84	−99.58
Ascorbic Acid	−8.56	−8.77	−8.69	−8.96	−9.09	−8.81	−88.26

**PL^pro^**							
Methotrexate	−11.99	−11.99	−11.99	−11.99	−12.00	−11.99	−80.26
Galidesivir	−12.36	−11.39	−12.06	−11.03	−12.68	−11.90	−126.95
Pralatrexate	−11.10	−11.25	−11.54	−12.02	−12.24	−11.76	−92.36
Mefloquine	−10.84	−10.76	−10.76	−10.76	−10.76	−10.78	−83.26
Daunonubicin	−9.42	−9.04	−8.98	−10.06	−11.06	−9.71	−82.06
Itraconazole	−8.95	−8.78	−9.06	−10.25	−10.17	−9.44	−69.20

**Methyl transferase**							
Methotrexate	−8.57	−8.98	−9.26	−8.98	−9.58	−9.07	−90.58
Vinadarabine	−8.56	−9.12	−8.56	−8.69	−8.77	−8.74	−65.89
Saquinavir	−8.36	−8.55	−8.45	−8.60	−9.02	−8.60	−92.05
Capreomycin	−9.78	−9.68	−10.06	−8.89	−9.08	−9.49	−94.28
Viomycin	−7.85	−7.89	−8.26	−8.65	−8.79	−8.29	−100.60
Saralasin	−8.29	−8.88	−8.76	−8.23	−7.26	−8.28	−95.28

**Nucleocapsid**							
Nelarabine	−8.51	−8.56	−8.40	−9.02	−9.14	−8.73	−90.58
Montelukast	−8.87	−9.05	−9.50	−9.56	−9.68	−9.33	−84.35
Paclitaxel	−8.20	−8.54	−8.64	−8.42	−8.74	−8.51	−77.01
Quinaprilat	−8.75	−8.79	−7.95	−8.40	−8.51	−8.48	−94.02
Regadenoson	−8.95	−8.05	−8.74	−8.09	−7.99	−8.36	−65.84
Bromfenac	−8.41	−8.96	−8.10	−7.58	−8.74	−8.36	−84.85

$ Docking score in individual snapshots generated from molecular dynamics (F1-F5)

* Average docking score.

# Average MMGBSA score.

### 3.3 Drugs targeting multiple SARS-CoV2 proteins

**Fig. 10:**
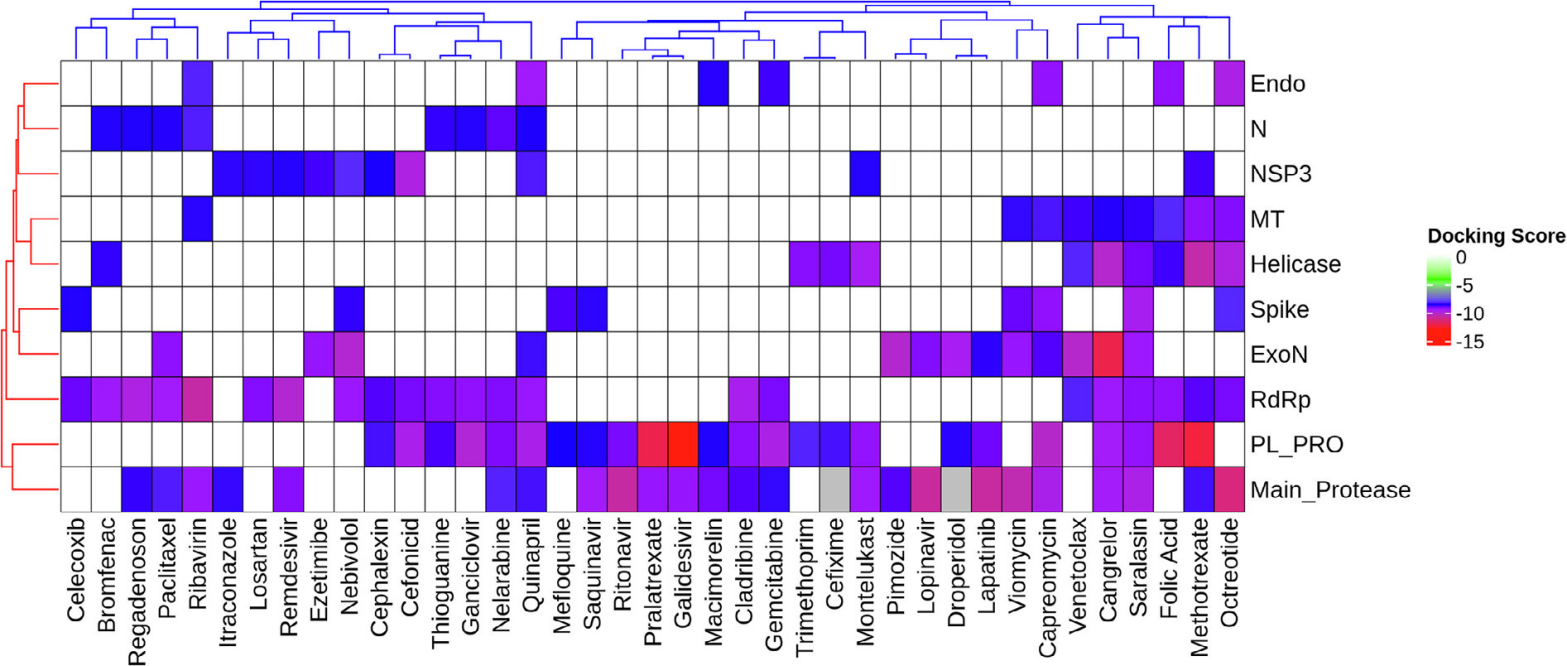
Drugs binding to multiple proteins. The drugs are shown along the X-axis while the proteins are shown along Y-axis. The white-red colour spectrum shows increasing binding affinity. (For interpretation of the references to colour in this figure legend, the reader is referred to the web version of this article.)

A heatmap (Fig. 10) was generated using the docking scores to summarize the binding of important drugs to multiple proteins. Individually or in combinations these drugs can serve as potential therapeutics with the capacity to modulate both the viral as well as human proteins. The identification of molecules targeting multiple viral proteins simultaneously will effect the virus life cycle at multiple stages and will also have a higher barrier towards evolution/emergence of drug resistant mutants, a common problem with many direct acting antivirals (DAA) against RNA viruses. Using a combination of drugs that target different viral proteins we can achieve synergistic antiviral effect. The detailed list of drugs and their docking scores is given in Supplementary Table 1.

### 3.4 Analysis of the transcriptome data and drug interactions of few differentially expressed genes

As stated earlier, the differentially expressed genes (DEGs) were obtained from the data reported by Blanco-Melo *et al* [35]. The DEGs were selected based on the following criteria: |log_2_FC|≥1, P-value ≤ 0.01 (Supplementary Table 2). This criteria was chosen to select genes showing the most significant variation. The gene ontology (GO) enrichment analysis on these DEGs indicates immune system processes, such as type II interferon signaling (IFNG), innate defence response, cytokine and chemokine siganling, RAGE receptor binding, secretory granule are among the most enriched ontologies (Supplementary Table 3). It is important to note that these are among the processes usually activated in infection-associated inflammation. Protein-protein-interaction network analysis was done using Cytoscape to identify the network hubs based on their interactions with other proteins. The giant component was extracted from the network with 1823 nodes containing 2546 interactions. Top 5% of the proteins (total 91) were selected based on the connections they make to other nodes in the network for further analysis (Supplementary Table 4).

**Fig. 11:**
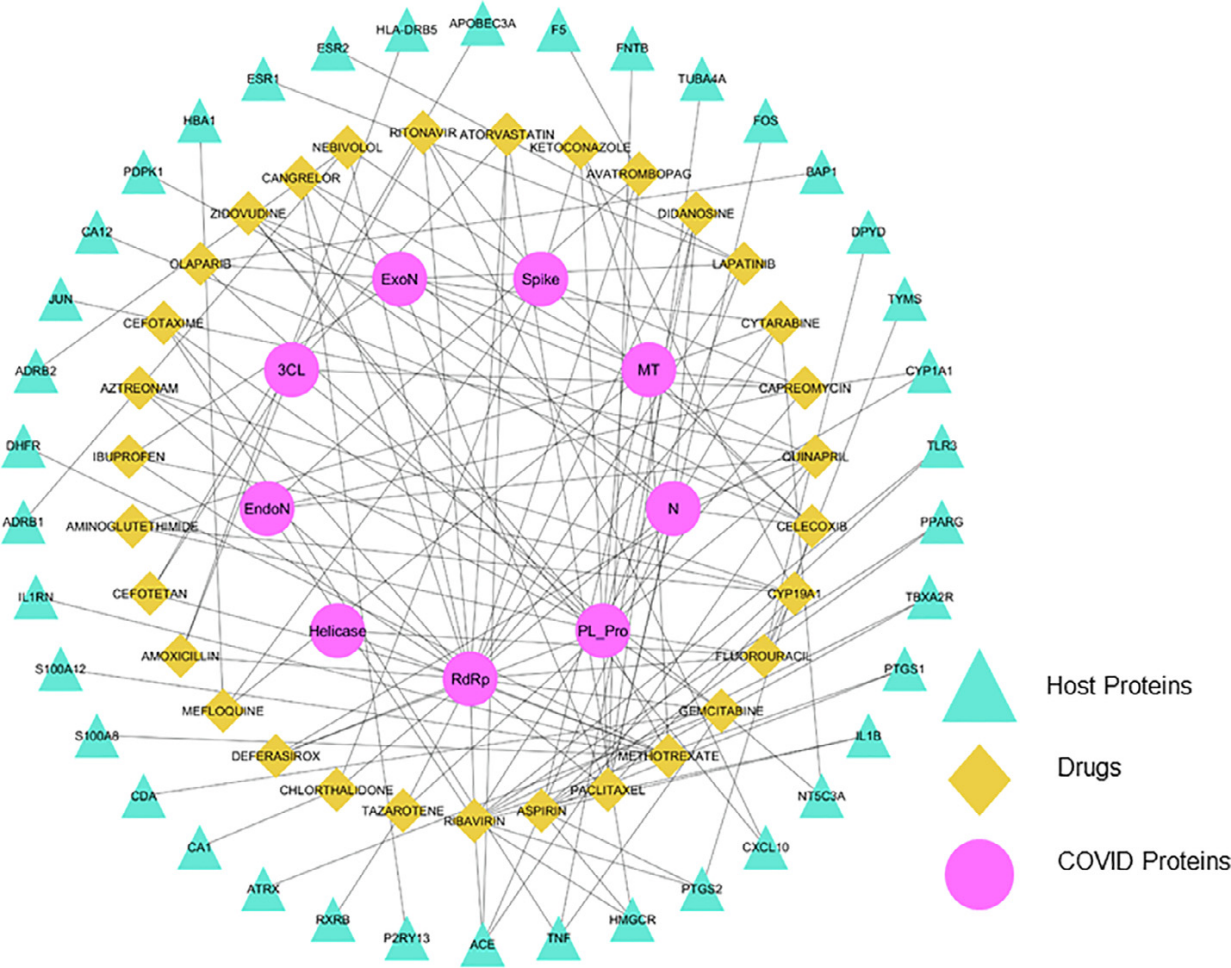
Human host and SARS-CoV2 protein drug interactions. Drugs capable of binding to multiple viral as well as human proteins related with SARS-CoV2 infection. The pink circles show the name of the SARS-CoV2 proteins, the green squares depict the drugs and the triangles show the name of the human proteins. The lines show the interaction between drugs and target protein. (For interpretation of the references to colour in this figure legend, the reader is referred to the web version of this article.)

It is worth to mention that among the human proteins many of them (e.g. ARRB2, JUN, CDC73, SUMO, TNFα, IL2RG, MCM7, IFIT1, FOS and ISG15 etc.), with prominent role in inflammation and immune response, are hubs i.e. very important proteins in the generated protein–protein-interaction network. A search for the selected DEGs (292) at drug-gene interaction database (DGIdb) resulted in the identification of 658 unique drugs for 97 proteins (Supplementary Table 5). An intersection of these with drugs binding with viral proteins (docking score ≤ −8.5) resulted in identification of 74 drugs that can target at least one viral protein whereas there are 31 drugs that can target atleast two viral proteins and one or more human proteins differentially expressed as a result of SARS-CoV2 infection. Recently, Li et al. [107] have reported a set of drugs for repurposing in COVID-19 using analysis of transcriptomic data of human tissue samples. The study identified a list of monoclonal antibodies along with FDA approved drugs. There are many drugs such as methotrexate, indinavir, chloroquine, sequinavir, and ritonavir, that are common between our study and the said study further corroborating our findings. It is interesting to find many drugs with multi-targeting ability against hub proteins as well as SARS-CoV2 proteins. Such drugs can have a significant therapeutic utility for COVID-19 (Fig. 11).

### 3.5 *In-vitro* validation of identified high affinity binders

We assessed the activity of few of the compounds that bind to more than one SARS-CoV2 proteins in an *in-vitro* cell culture model of SARS-CoV2 in VeroE6 cells. We used the SARS-CoV2 virus (ILS-01) isolated from oropharyngeal swab sample of confirmed COVID19 positive patient.

**Fig. 12:**
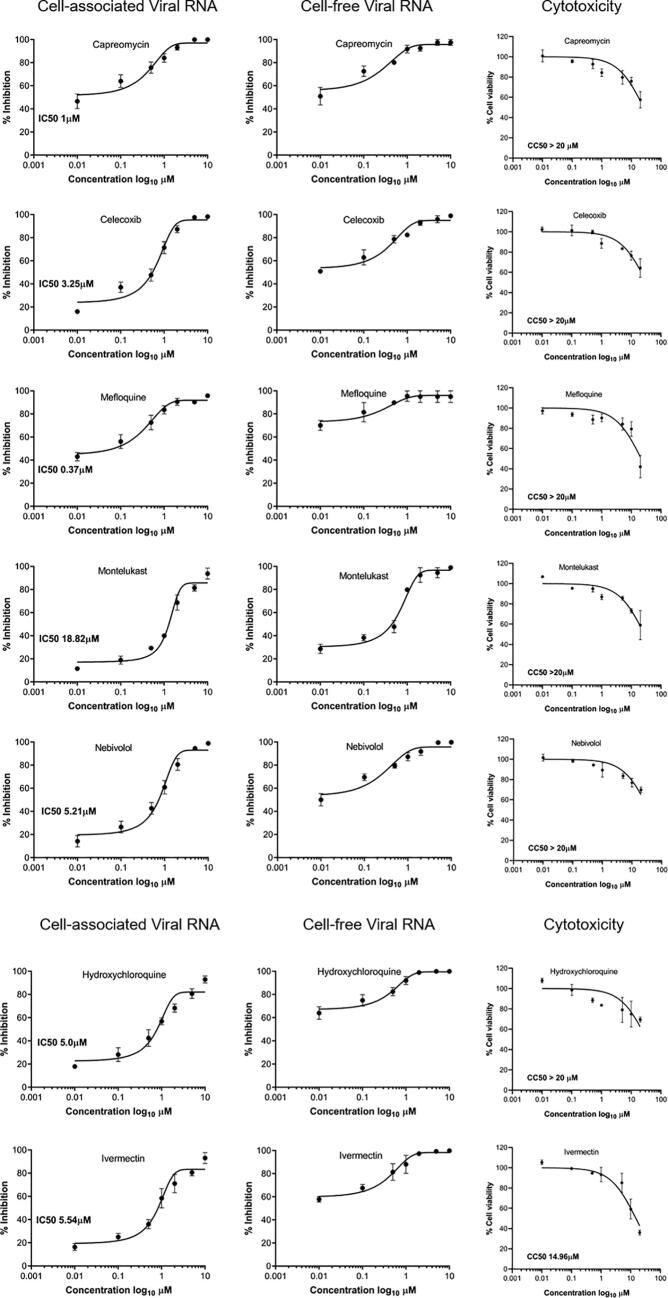
*In-vitro* validation of anti-SARS-CoV2 activity: Vero E6 cells were in infected with SARS-CoV2 at 0.1 MOI for 2 h. Subsequently the virus inoculum is replaced with fresh media containing the 0.1% of vehicle (DMSO) or indicated concentration (0.01, 0.1, 0.5, 1, 2, 5, 10 μM) of various drugs. 24 h post treatment the viral load in the cells (cell associated) or supernatant (secreted virus) was determined using real-time PCR as described in methods. IC_50_ values were determined from the dose response curve analysis (GraphPad prism). Vybrant MTT cell viability assay was used to determine cytotoxicity at low–high concentrations (0.01, 0.1, 0.5, 1, 5, 10 & 20 μM). The percentage cell viability was calculated with respect to vehicle treated control and 50% cytotoxic concentration (CC_50_) was determined by dose–response curve (GraphPad prism). Experiments were done in duplicates.

Seven different concentrations of the drugs ranging from 0.01 µM to 10 µM were used to determine their IC_50_ values. Almost all of the drugs tested showed anti-SARS-CoV2 activity further validating our in-silico analysis. All of the compounds showed more than 50% reduction in viral loads at minimal or non-toxic concentrations. Ivermectin, and hydroxycholoroquine, two of the compounds known to inhibit SARS-CoV2 replication *in-vitro,* were used as positive controls in this study that further validated the activity and potency of the molecules tested in this study. Among the 5 drugs tested mefloquine has the lowest IC_50_ value at 0.37 uM and montelukast, which was also predicted by others to possess anti-SARS-CoV2 potential has a higher IC_50_ value of 18.82 uM (Fig. 12, Table 3).

**Table 3:** The activity (IC50) and cytotoxicity (CC50) of various drugs.

S. No.	Drug	IC50 (µM)	CC 50 (µM)
1	Capreomycin	1	>20
2	Celecoxib	3.25	>20
3	Mefloquine	0.37	>20
4	Montelukast	18.82	>20
5	Nebivolol	5.21	>20
6	Hydroxychloroquine	5	>20
7	Ivermectin	5.54	14.96

To further validate the anti-SARS-CoV2 potency of the tested drugs we performed an immunofluorescence assay by staining for SAR-CoV2 nucleocpasid protein in SARS-CoV2 infected Vero-E6 cells subjected to treatment with the vehicle control or drugs at their IC50 concentrations.

**Fig. 13:**
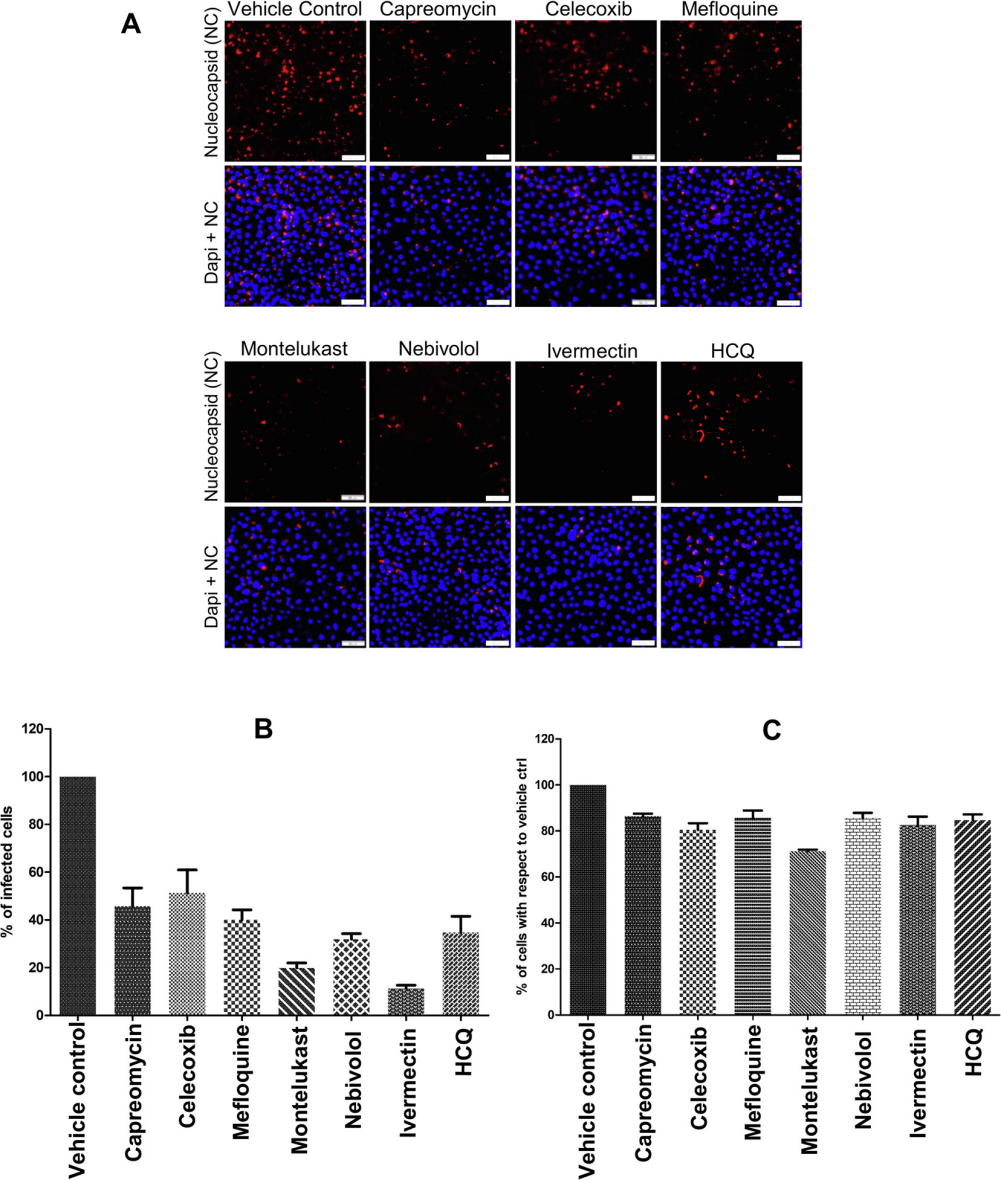
Immunofluorescence assay for validating the anti-SARS-CoV2 potency of the identified drugs. (A) Immunofloresence images of SARS-CoV-2-infected Vero E6 cells stained with antibody against SARS-CoV2 nucleocapsid. (B) Graphical representation of the percent infected cells with respect to vehicle control. (C) Graphical representation of percent total number of cells with respect to vehicle control. Cells were counter stained with Dapi nuclear stain. The experiment was performed in triplicates and the data presented here is the mean ± SEM. Hydroxycholoquine is represented as HCQ.

We observed marked reduction (around 50% or higher) in the percentage of infected cells subjected to treatment with the drugs in comparison to the vehicle control (Fig. 13A and B). We also observed that the drug treatment at the indicated IC50 concentrations had very minimal effect on the total cell number in comparison to the vehicle control (Fig. 13C). Overall these observations suggest that the treatment with drugs resulted in marked inhibition of viral gene expression with minimal effect on cellular viability.

Overall these observations strongly validate our *in-silico* finding, however further screening is required in physiologically relavant cell lines and in-vivo animal models to fully establish the anti-SARS-CoV2 potential of the identified leads.

## 4 Discussion

The computational drug repurposing studies came into forefront bacause of the speed and memory of the modern computers supplemented by the availability of the algorithms and data from studies in the past. Moreover, the crystal structures of many SARS-CoV2 proteins were reported during 2020 that made the structure based screening studies feasible. Various libraries (e.g. FDA approved) were screened against targets like RdRp, main protease (3CLpro), spike protein, membrane protein, and non-structural proteins (Nsps) using various strategies [74], [108], [109], [110], [111], [112]. For example, the main protease (Mpro) was screened by three docking algorithms. The authors selected the molecules that are commonly predicted by all algorithms [108]. Similarly, structure based docking followed by molecular dynamics studies have been used by Al-Khafaji et al. [113], Mittal et al. [114] and Wang et al. [111]. Wu et al. [74] screened ZINC database molecules against a number of SARS-CoV2 and host proteins. A set of antiviral drugs was screened for their possible activity against 5 SARS-CoV2 proteins using deep learning [115]. Zhou et al [116] identified set of molecules against SARS-CoV2, using a network proximity analysis combining information from HCoV-host interactions, and human protein interactome.

The strategy of combining multiple approaches (gene expression, graph based algorithms, docking and molecular dynamics etc.) and targeting both the SARS-CoV2 and human proteins is the novelty of this study. The overall goal was to identify molecules that can bind with multiple SARS-CoV2 proteins that play vital role(s) in various stages of the viral lifecycle as well as target the host factors that drive viral persistence and disease pathogenesis. Our analysis predicted drugs that can bind to viral proteins (both the structural and non-structural proteins) with high affinity and can effectively inhibit viral entry as well as the post entry events like viral genome replication and transcription.

Capreomycin is a promising candidate with potential to inhibit SARS-CoV2 at mutltiple stages of viral lifecycle, as it can bind with high affinity to spike protein and the viral proteases and methyl transferase, which play a crucial role in viral entry, replication and transcription. In our *in-vitro* asays it has shown good inhibitory activity.

Mefloquine, an antimalarial drug, has shown good affinity towards spike protein in our *in-silico* studies. It is pertinent to note that recently mefloquine has been shown to prevent the entry of SARS-CoV-2 into host cells. It has shown potent inhibitory activity against SARS-CoV2 in multiple cell lines [117]. Many other studies reported similar observations corroborating our findings [109], [112], [118].

Our analysis further indicated that some drugs that bind viral proteins also target some of host proteins that are differentially expressed in lung tissue during SARS-CoV2 infection. The predicted drug candidates that interact with the viral protein(s), in parallel can also specifically target the host signalling pathways vital to control viral infection or disease manifestation. For instance, nebivolol a β-adrenoreceptor blocker, which stimulates nitric oxide production by endothelial nitric oxide synthase [48] is found to bind to PL^pro^ and exonuclease of SARS-CoV2. Nitric oxide is used to reverse pulmonary hypertension and shown to improve severe hypoxia in SARS-CoV1 [119] and SARS-CoV2 patients. Hence, nebivolol can be a promising therapeutic strategy with dual benefit; i) to curb SARS-CoV2 infection and ii) reversal of severe hypoxia manifestation in critical Covid-19 patients via its direct effect on nitric oxide synthase. Corroborating our findings, another recent study also reported that nebivolol can efficiently inhibit SARS-CoV2 in submicromolar range [120].

A major contributor of COVID-19 pathology is hyper-inflammation and cytokine hyperactivity. Strategies to reduce the inflammation and cytokine hyperactivity have shown promising results. Celecoxib, a selective cyclooxygenease-2 (COX-2) inhibitor, which lowers the effect of proinflammatory cytokines IL-6 and IL-1β [121], can also target Spike and RdRp protein of SARS-CoV2. Thus administrating celecoxib to COVID-19 patients can have dual benefit of reducing systemic inflammation as well as inhibition of viral replication. It has been shown that COX inhibitors can inhibit viral replication and production of virus particles in other coronaviruses [122]. A recent review [123] suggested selectively targeting COX2 and closely related cascades could be significant in the treatment of COVID-19. The authors were of the opinion that celecoxib has potential and should be evaluated clinically.

The leukotriene inhibitor montelukast is shown to reduce proinflammatory cytokines e.g. TNF-α, IL-6 and IL-1β levels [124], [125]. A previous study suggests that it inhibits Zika virus by disrupting the integrity of the virions [126]. Durdagi et al. [127] using a multiscale modeling approach and in-vitro evaluation identified it to interefere with viral entry through Spike-ACE2 interface and by inhibiting the main protease. Apart from the anti-asthmatic effect it is also reported to cross BBB and reduce neuroinflammation [128]. A recent paper reviewed its antiviral, anti-inflammatory, anti-allergic and anti-fibrotic activities. It has also been suggested that montelukast should be tried as therapeutic option in COVID-19 [129].

Interestingly, the drugs montelukast, celecoxib and nebivolol can cross blood brain barrier [128], [130], [131], which gives additional advantage to counter neurological manifestations of COVID-19. It is again pertinent to note that these drugs have shown appreciable inhibitory activity (IC_50_) against SARS-CoV2 in our study.

Lapatinib binds to Nsp14, a viral protein crucial for viral RNA synthesis [132]. Lapatinib is a HER2 inhibitor, which can also trigger TBK1 activation that plays a crucial role in anti-viral signalling. Computational studies have predicted lapatinib to be able to bind many SARS-CoV2 proteins including the main protease [110], [133]. Thereby, lapatinib has the dual advantage of inhibiting SARS-CoV2 replication as well as upregulating anti-viral signaling [134], [135], [136].

Saralasin belongs to a class of drugs called angiotensin receptor blockers (ARBs). It is worth mentioning some other ARBs (e.g. losartan) are also in clinical trials as therapeutics for COVID-19 (https://clinicaltrials.gov, IDs: NCT04287686, NCT04312009, NCT04311177). Moreover, it is reported to bind to many other SARS-CoV2 targets [137], [138]. Therefore, it is also a good candidate worth considering.

Similarly, bronchodialator indacaterol, which targets the exonuclease is also a promising agent due to its ability to regulate genes involved in suppressing pro-inflammatory cytokine production and attenuation of immune response [139], [140], [141]. Another study has reported indacaterol to be able to bind spike protein of SARS-CoV2 [142].

The transcription complex activator protein 1 (AP1) is composed of homo/hetero dimers of Fos, Jun, CREB and other activated transcription factors (ATFs). The studies on the SARS-CoV1 infection in the Vero and Huh7 cell shows that nucleocapsid protein is the potent activator of (AP-1) [143]. Interestingly, asthmatic patients show higher expression of c-Fos in their epithelial cells. It is also observed that TNF-α induced ROS and intracellular glutathione depletion in the airway epithelial cells induces the production of AP-1 and leads to the pulmonary fibrosis [144], [145]. Our analysis suggests that paclitaxel and bromocriptine, which dock with nucleocapsid and Nsp4 proteins can also effectively bind to c-Fos and thereby would be beneficial in inhibiting SARS-CoV2, as well as in alleviating lung injury observed in COVID-19 patients. Interestingly, other groups have also reported that bromocriptine is able to bind to main protease [146] or Nsp14 [147].

The transcriptome analysis revealed that S100/calgranulin is upregulated during SARS-CoV2 infection. This protein is also found in higher quantity in the Bronchoalveloar Lavage Fluid (BALF) and sputum of patients with inflamed lungs, COPD, and ARDS [148]. Calgranulin is polypeptide released by the activated inflammatory cells such as leukocytes, PBMC phagocytes and lymphocytes and is accumulated at the sites of chronic inflammation. It is the ligand for RAGE receptors and is the major initiator of cascading events that amplify inflammation [149]. Our analysis suggests that the anti-inflammatory agent methotrexate which has high affinity to the Nsp16 protein of SARS-CoV2 also shows appreciable binding to calgranulin and can thereby be useful to curtail systemic inflammation observed in lungs during COVID-19 in addition to its inhibitory effect on SARS-CoV2. Interestingly, methotrexate is recently reported to inhibit the replication of SARS-CoV2 [150]. Another paper suggested to use methotrexate with leucovorin rescue for the treatment of severe COVID-19 [151]. Methotrexate is also in clinical trial currently for the treatment of mild COVID-19 (https://clinicaltrials.gov/ct2/show/NCT04610567).

The expression of endogenous prolactin is also upregulated during SARS-CoV2, which leads to prolactin induced STAT5 activation and its pathways. Prolactin has a dual role in human physiology functioning as a hormone (secreted from anterior pituitary gland) and cytokine (secreted by immune cells). It causes anti-apoptotic effect and induces proliferation in immune cells in response to antigens leading to increased production of immunoglobulin, cytokines, and autoantibodies [152]. We envisage that prolactin may be one of the significant player in trigger of cytokine storm implicated in COVID-19. Interestingly, our study suggests that zidovudine, which targets O’-methyl transferase (Nsp16) can also bind to prolactin and can be of high significance in management of COVID-19 due to dual ability to affect Nsp16 and prolactin.

The COVID-19 creates an inflammatory state involving proinflammatory cytokines e.g. IL-6, TNF-α etc. IL-6 stimulate ferritin and hepcidin synthesis [153]. The hyper-ferritinemia is associated with generation of ROS and RNS leading to enhanced systemic inflammation. As a result a devastating cycle is propogated where increased ferritin leads to higher inflammation (increased IL6) resulting in further increase in ferritin levels [154]. Hyper-ferritinemia has been linked with poor prognosis in COVID-19 patients, evidenced by high levels of ferritin in non-survivors as compared to survivors [155], [156]. In this milieu, iron chelators can be extremely helpful by reducing the hyper-ferritinemia and systemic inflammation. Deferoxamine is an iron chelator that also increases degradation of ferritin by lysosomes leading to reduction of free radicals and subsequently inflammation. It also limits the chances of ARDS and tissue fibrosis. Our analysis indicates that deferoxamine binds to RdRp and PL^pro^ protein of SARS-CoV2 with good affinity. Therefore it can be a good candidate for the therapeutics of COVID-19. Currently, desferaox is under clinicl trials for the treatment of COVID-19.

We could test a few molecules that showed potent anti-SARS-CoV2 activity in *in-vitro* models. The identified molecules are commonly used drugs and hence can be quickly repurposed. Their combinations can also have synergistic effcts against SARS-CoV2. We hope these molecules will prove to be useful in our fight against COVID-19. Apparently, the cytotoxicity of some molecules is high. As the in-vitro assays were performed in Vero-E6 cells, a monkey kidney epithelial cell line, we expect that the cytotoxicty may be lower in human cell lines. However, they are FDA approved drugs already being used for treating various clinical conditions at the recommended dosage and may be benefical for SARS-CoV2 therapeutics. Also the treatment for SARS-CoV2 will likely last only for a short duration, therefore weighing the potential benefits vs toxicity they could be very useful in curbing SARS-CoV2 infection.

## 5 Conclusions

Currently, there are no approved coronavirus treatments and therefore there is a pressing need for drugs that can be effective therapeutics for COVID-19. Our study predicted promising drug candidates with high binding affinity towards many of SARS-CoV2 proteins. These drugs are expected to be more effective than drugs that target single viral proteins due to their ability to affect multiple aspects of viral lifecycle and enhance the barrier towards the evolution of drug-resistant mutants, a usual phenomenon observed in RNA viruses.

Overall our study predicts promising agents with potential to inhibit crucial viral processes, upregulate anti-viral host response and alleviate severe lung disease condition thereby providing attractive avenues for design of potential and multipronged therapeutic strategies against COVID 19.

## Author Contribution

The work is designed and conceptualized by SK, AD, GA and GHS. Data generation and work performed by SK, PVK, PK, BS and AD. The manuscript was prepared by SK, AD, PK, GA, PVK, BS, TKB and GHS.

## Declaration of Competing Interest

The authors declare that they have no known competing financial interests or personal relationships that could have appeared to influence the work reported in this paper.
