# Supplementary material for: Identification of multipotent drugs for COVID-19 therapeutics with the evaluation of their SARS-CoV2 inhibitory activity
Source: Comput Struct Biotechnol J. 2021 Apr 7;19:1998–2017. doi: 10.1016/j.csbj.2021.04.014 (PMC8025584; doi:10.1016/j.csbj.2021.04.014)
Supplement: Supplementary data 1 [file mmc1.zip › Supplementary_methods_Covid.docx]

**Identification of multipotent drugs for COVID-19 therapeutics with the evaluation of their SARS-CoV2 inhibitory activities**

Sugandh Kumar^1,2,#^, Bharati Singh^1,2,#^, Pratima Kumari^1,4^, Preethy V Kumar^1,2^, Geetanjali Agnihotri^3^, Shaheerah Khan^1,4^, Tushar Kant Beuria^1^, Gulam Hussain Syed^1*^ and Anshuman Dixit^1*^

^1^Institute of Life Science, Nalco Square, Bhubaneswar, Odisha, India-751023.

^2^School of Biotechnology, Kalinga Institute of Industrial Technology (KIIT) University, Bhubaneswar, Odisha, India-751024.

^3^School of Chemical Technology, Kalinga Institute of Industrial Technology (KIIT) University, Bhubaneswar, Odisha, India-751024.

^4^Regional Centre for Biotechnology (RCB), 3rd Milestone, Faridabad-Gurgaon, Haryana 121001, India

^*^**Corresponding author**

**# Equal Contribution**

**Contact Information:** E-mail: [anshumandixit@ils.res.in](mailto:anshumandixit@ils.res.in), [gulamsyed@ils.res.in](mailto:gulamsyed@ils.res.in).

Institute of Life Sciences, Nalco Square, Bhubaneswar, 751023, Odisha, India; Tel: +91-674-230-0137.

**Supplementary methods:**

**Sec. 1.1: Molecular dynamics simulation system setup**

Each SARS-CoV2 proteins was prepared for the simulation in VMD v1.9.3 (www.ks.uiuc.edu/Research/vmd). Briefly, each receptor was prepared using autopsf and then solvated in a rectangular TIP3P water box with 10 Å buffering distance. These water molecules were adopted using SETTLE algorithm. To ensure electro-neutrality Na+ and Cl- ions were added to each of the system. Simulations were performed using the NAMD 2.6 on a high performance linux cluster using 250 processors. Each of the systems was subjected to stepwise minimization and equilibration process. The water molecules, ions, and protein sidechains were minimized for 40,000 steps by keeping the protein backbone atoms fixed. Further, the structures were relaxed with nominal restraints (i.e. 10 kcal/mol) on Cα atoms, to prevent any sudden conformational change in structure, for 40,000 steps. Thereafter, each system was gradually heated (0 to 310 K in steps of 30 K) with a canonical ensemble (NVT) and at each step 20 ps simulation was performed to let the system adjust with temperature. At 310 K, an isobaric and isothermic ensemble (NPT) was applied for 100 ps by keeping a constant pressure of 1.0 bar using the Langevin dynamics. Further, all restraints were removed and each system was subjected to 1 ns simulation using Langevin piston coupling algorithm. During the entire MD simulation, a time step of 2 fs was applied by keeping the hydrogen bond lengths fixed. The long-range electrostatic interactions were handled using Particle Mesh Ewald (PME) algorithm with fixed periodic boundary conditions. The SHAKE algorithm was used to handle bonds to hydrogens. After the completion of the equilibration process, a 20 ns production run was performed.

**Sec. 1.2: Molecular Docking**

The FDA-approved drug library was used for virtual screening. Before the screening, all drugs were prepared by ligprep module of Maestro, Schrödinger 9.3 with default settings. Prior to docking, the proteins were prepared by the addition of missing atoms/residues, addition of hydrogens, and charges. The protein structure was optimized by energy minimization using “protein preparation wizard”. The docking grid was generated by selecting the residues within 5Å from the co-crystallized ligand. The glide module with standard precision (SP) mode was used for docking with the flexible ligand sampling method. The co-crystallized ligands were docked first to check the efficiency of docking by comparing the RMSD of the docked pose with co-crystallized conformation. The docking of FDA approved library was done once the co-crystallized pose was regenerated (RMSD<2Å). The best candidate ligands were selected based on the docking score. The docking poses were subjected to MMGBSA analysis. The final ranking of the molecules was obtained by calculating the average glide score in the five snapshots of a viral protein generated by molecular dynamics simulation to include the effect of binding site dynamics.


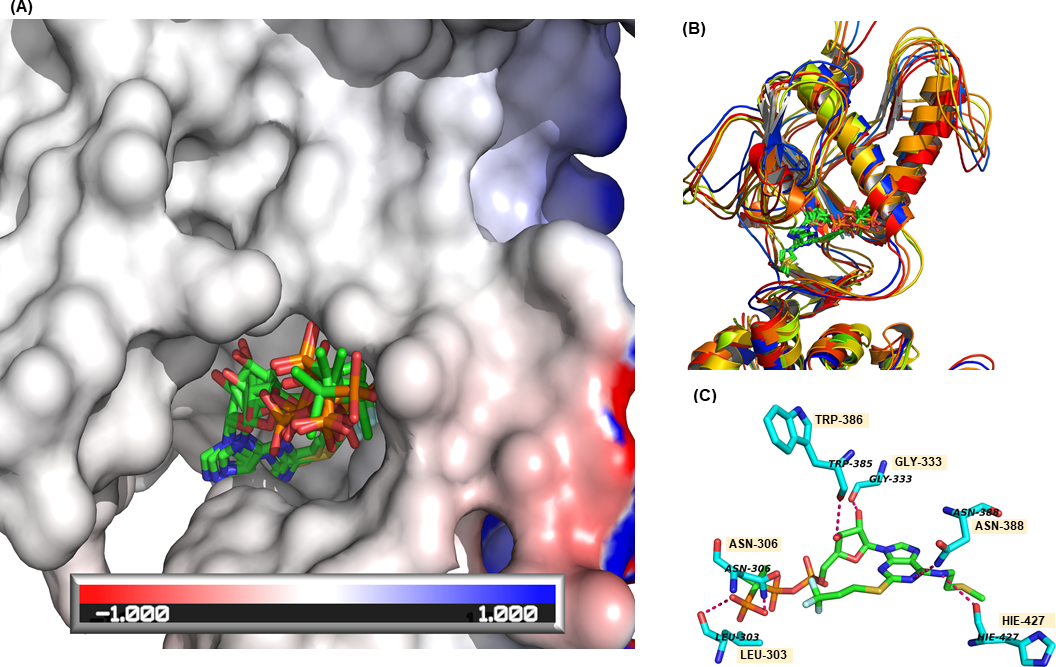


**Supplementary Figure 1:** **Cangrelor binding to ExoNulease:** (A) The surface view of the protein and cangrelor binding. (B) The MD frames showing the flexibility of the active site and binding of ligand in the MD snapshots (average docking score -11.15) (C) The hydrogen bonding interactions of cangrelor with ExoNuclease protein.

**
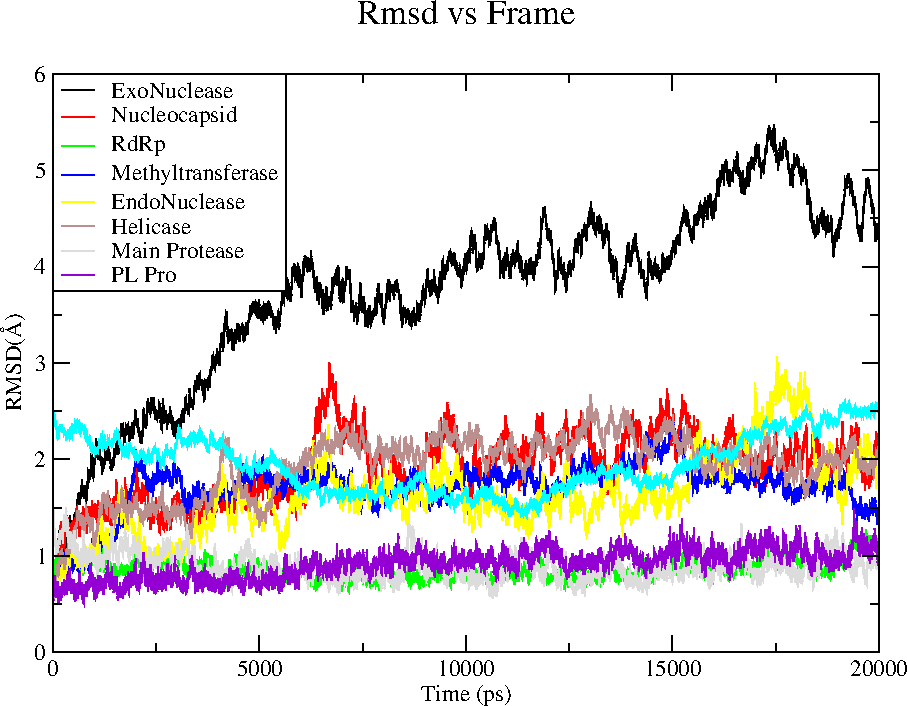
**

**Supplementary Figure 2:** **Root mean square deviation (RMSD).** The RMSD curves show that the molecular dynamics simulations got stabilized very quickly (<4ns) and were stable throughout the simulation as evidenced by small range (<2Å) bound movement of the RMSD curves.


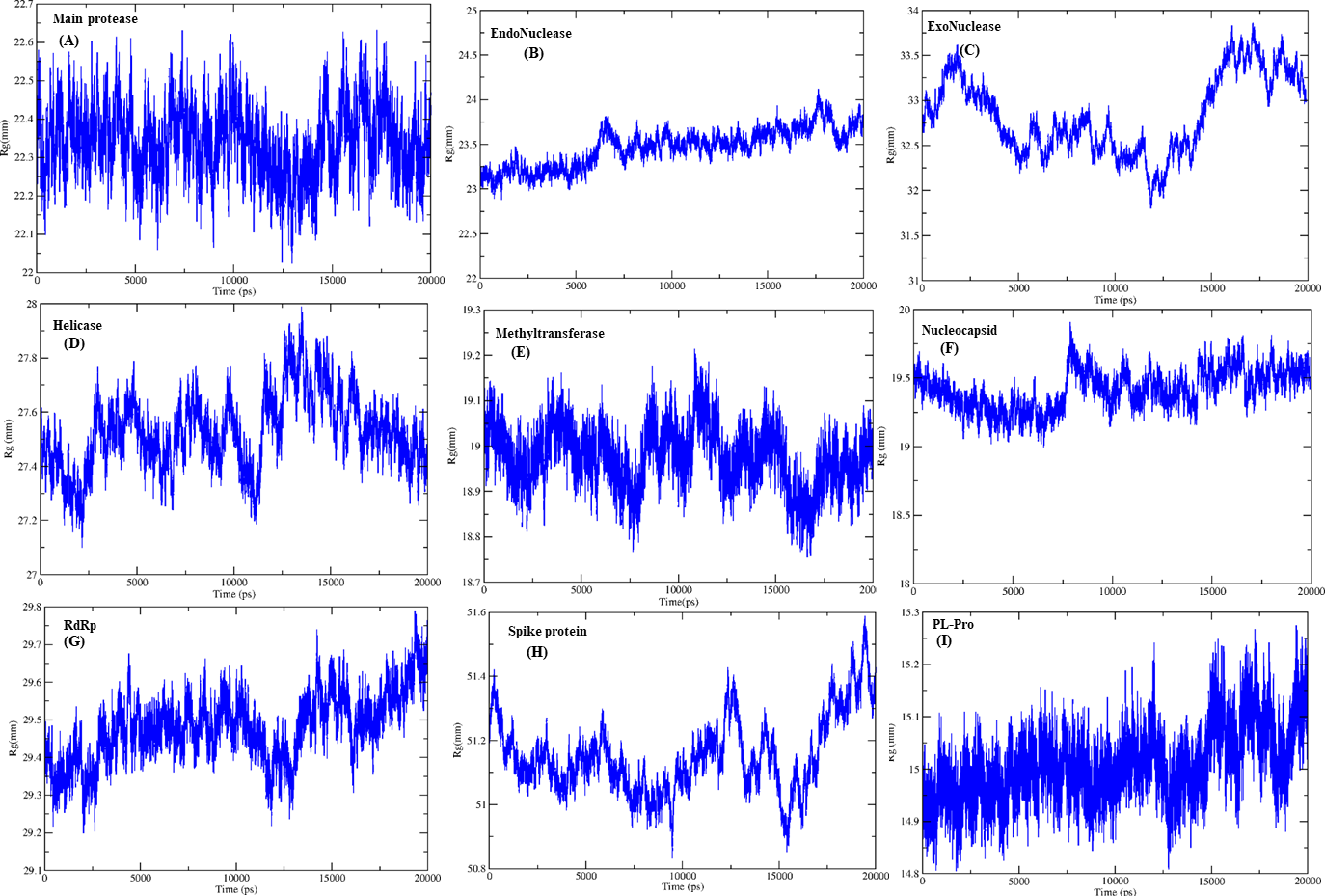


**Supplementary Figure 3:**  **Radius Of Gyration (ROG)** (A-I) was calculated to check the compactness of the protein. The ROG curves further indicate the stability of the conformations during the simulations.
