# Supplementary figures and images for: Identification of multipotent drugs for COVID-19 therapeutics with the evaluation of their SARS-CoV2 inhibitory activity
Source: Comput Struct Biotechnol J. 2021 Apr 7;19:1998–2017. doi: 10.1016/j.csbj.2021.04.014 (PMC8025584; doi:10.1016/j.csbj.2021.04.014)

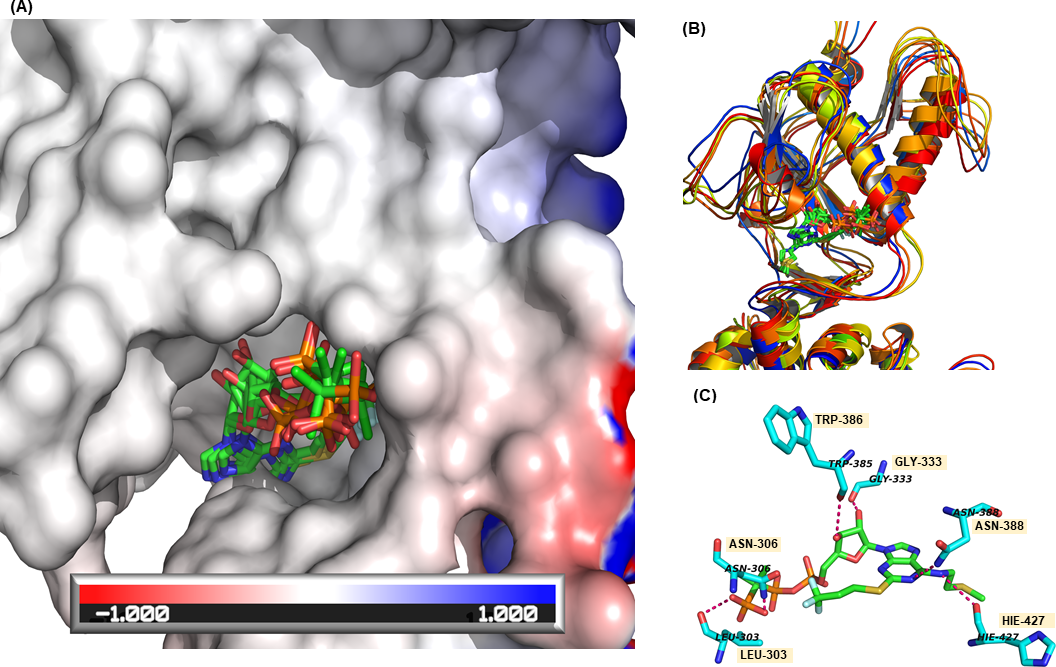

Supplement: Supplementary data 1 [file mmc1.zip › Supplementary Fig. 1.tif]

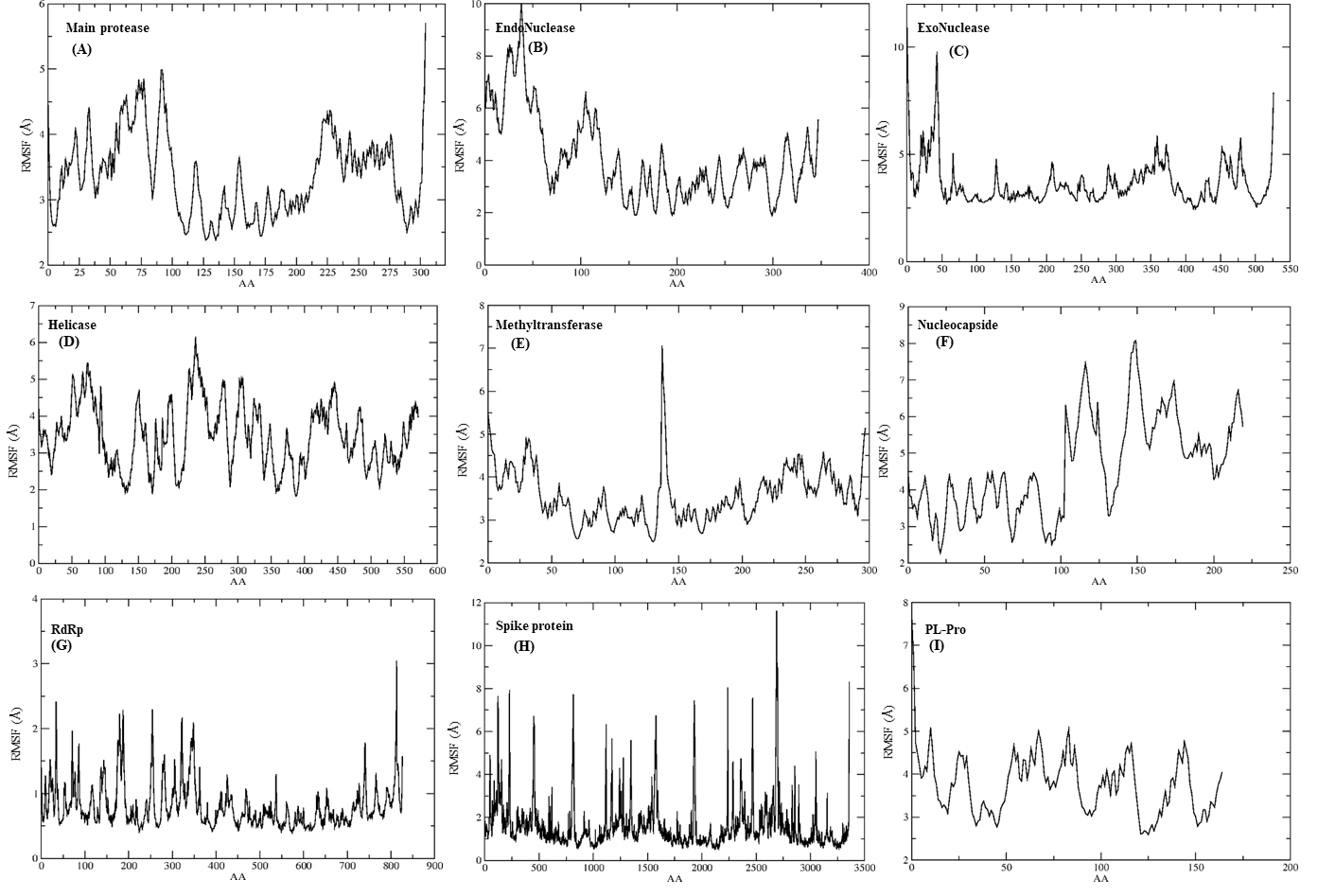

Supplement: Supplementary data 1 [file mmc1.zip › Supplementary Fig. 2.tif]

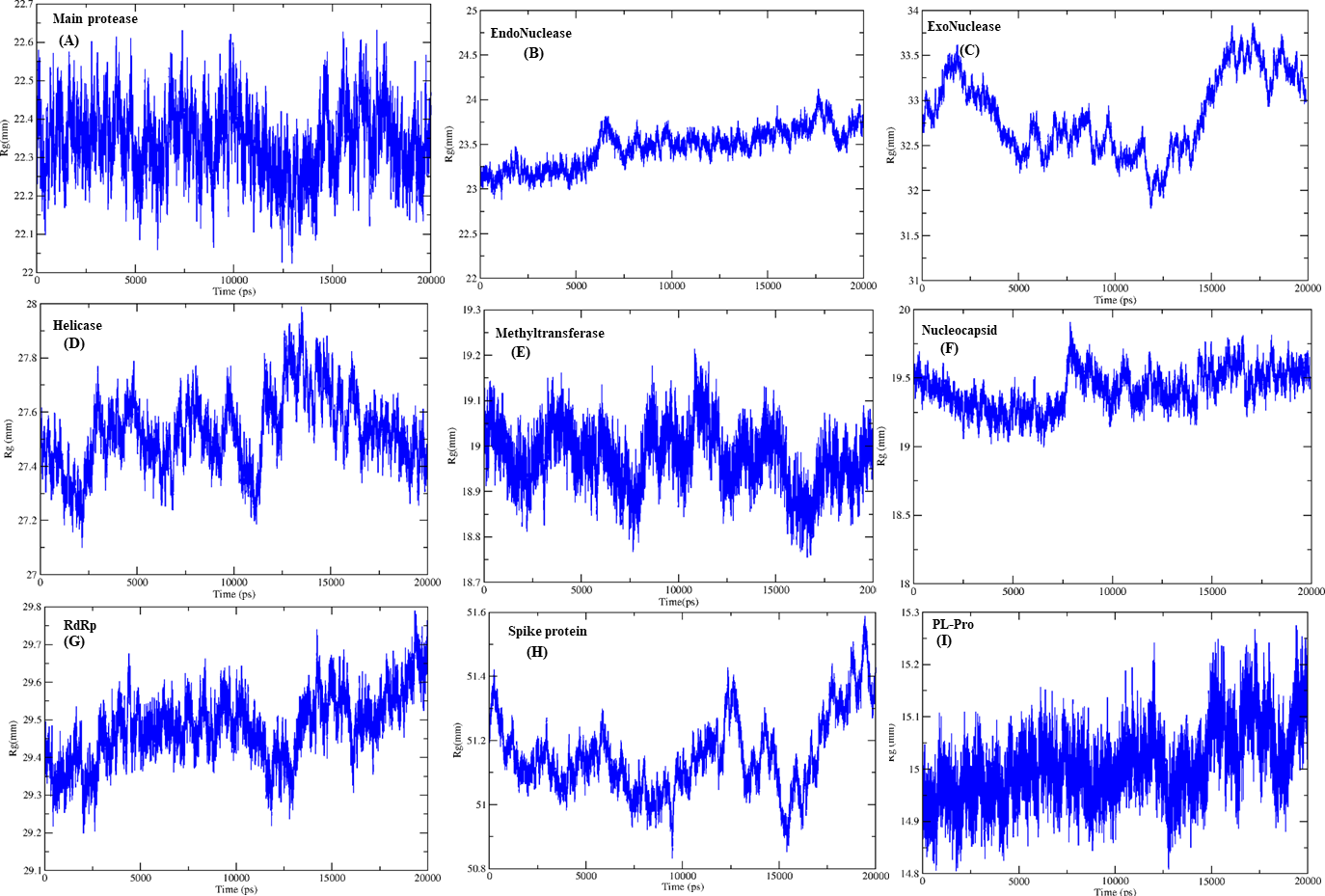

Supplement: Supplementary data 1 [file mmc1.zip › Supplementary Fig. 3.tif]
